# Myocardial Aldosterone Receptor and Aquaporin 1 Up-Regulation Is Associated with Cardiomyocyte Remodeling in Human Heart Failure

**DOI:** 10.3390/jcm10214854

**Published:** 2021-10-22

**Authors:** Andrea Frustaci, Claudio Letizia, Cristina Chimenti, Romina Verardo, Maria Alfarano, Rossella Scialla, Giulia Bagnato, Fabio Miraldi, Luigi Sansone, Matteo Antonio Russo

**Affiliations:** 1 Department of Clinical, Internal, Anesthesiologist and Cardiovascular Sciences, Sapienza University, 00161 Rome, Italy; cristinachimenti@libero.it (C.C.); maria.alfarano@uniroma1.it (M.A.); fabio.miraldi@uniroma1.it (F.M.); 2 Cellular and Molecular Cardiology Lab, IRCCS L. Spallanzani, 00149 Rome, Italy; romina.verardo@inmi.it (R.V.); rossella.scialla@inmi.it (R.S.); giulia.bagnato@inmi.it (G.B.); 3 Department of Translation Medicine and Precision, Sapienza University, 00161 Rome, Italy; claudio.letizia@uniroma1.it; 4 Laboratory of Molecular and Cellular Pathology, IRCCS San Raffaele Pisana, 00163 Rome, Italy; luigi.sansone@sanraffaele.it; 5 MEBIC Consortium, San Raffaele Open University, 00163 Rome, Italy; matteoantoniorusso44@gmail.com; 6 IRCCS San Raffaele Pisana, 00163 Rome, Italy

**Keywords:** hyperaldosteronism, myocardial damage, aldosterone receptor, vacuolar degeneration, cellular and molecular rehabilitation

## Abstract

Background: Abnormal aldosterone signaling is a recognized source of cardiovascular damage. Its influence on cardiomyocyte structure, function, and hormonal receptors when associated with heart failure is still unreported. Methods: Twenty-six consecutive patients with heart failure (LVEF < 40%) and normal coronaries and valves underwent left ventricular endomyocardial biopsy (EMB) for evaluation of myocardial substrate. Biopsy samples were processed for histology, electron microscopy, immunohistochemistry, and Western blot analysis of myocardial aldosterone receptor and aquaporin-1 correlated with plasma aldosterone (AD) and renin activity (PRA). Eight patients with virus-negative inflammatory cardiomyopathy (ICM) had a control EMB after 6 months of immunosuppressive therapy and recovery of cardiac function with re-evaluation of cardiomyocyte structure and receptor expression. Results: EMB in addition to the diagnosis of myocarditis (15 cases), dilated cardiomyopathy CM (6), alcohol CM (2), and diabetic CM (3) showed vacuolar degeneration and cloudy swelling of cardiomyocytes corresponding at electron microscopy to ions and water accumulation into cytosol, membrane-bound vesicles, nucleus, and other organelles, and was associated with an increased AD, PRA, and myocardial expression of aldosterone receptor (2.6 fold) and aquaporin 1 (2.7 fold). In the 8 patients recovered from ICM, cardiomyocyte diameter reduced with disappearance of intracellular vacuoles and normalization of cytosol, nucleus, and cell organelles’ electron-density, along with down-regulation of aldosterone receptor and aquaporin-1. Conclusion: Human heart failure is associated with overexpression of myocardial aldosterone receptor and aquaporin-1. These molecular changes are paralleled by intracellular water overloading and cardiomyocyte swelling and dysfunction. Cardiac recovery is accompanied by down-regulation of hormonal receptors and normalization of cell structure and composition.

## 1. Introduction

The role of excessive aldosterone signaling in mediating cardiovascular damage is increasingly recognized by clinical and experimental evidence.

Convincing data come from Rales [1], Emphasis-HF [2,3], and Ephesus trials [4], where inhibition of myocardial aldosterone receptor (AR) has been followed by reduction in left ventricular hypertrophy and decrease in mortality and cardiovascular-related hospitalizations compared with placebo. As a consequence, aldosterone inhibitors are recommended by the current guidelines in the pharmacologic treatment of heart failure [5,6]. However, how myocardial expression of aldosterone receptor and its correlated aquaporin-1 (AQ1) modifies during heart failure and what impact it may have on myocyte structure and function is still unclear. A recent report on primary aldosteronism-associated cardiomyopathy [7] suggests an overexpression of AR and AQ1 to occur in cardiomyocytes and vessels’ smooth muscle cells with water accumulation in the cytosol and organelles causing cell swelling and dysfunction. These structural changes reverse with removal of hypersecreting adrenal adenoma and normalization of plasma aldosterone levels.

The aim of the present study was to evaluate the myocardial AR and AQ1 expression in human failing heart and after recovery and their influence on cell composition and function.

## 2. Methods

### 2.1. Patient Population

Twenty-six consecutive patients with dilated cardiomyopathy and left ventricular dysfunction (LVEF < 40%) who from January 2019 to December 2019 underwent a left ventricular endomyocardial biopsy study for morpho-molecular characterization of myocardial substrate represented our patient population [8]. At the time of enrollment, no patients were treated with aldosterone inhibitors. Among them, 15 patients received a diagnosis of virus-negative inflammatory cardiomyopathy, 6 had a non-familiar idiopathic dilated cardiomyopathy, 2 (with a history of addiction) had alcoholic cardiomyopathy, and the remaining 3 had a diabetic cardiomyopathy correlated with a long history of diabetes.

The patient population (group A) was compared with a control group of 30 patients with mitral stenosis and normal LV size and function undergoing surgical repair (group B). A total of 8 of 26 patients with an amenable disease (virus-negative active myocarditis) had a control biopsy after cardiac recovery, obtained following 6-months of immunosuppressive therapy according to the TIMIC protocol [9].

The study was approved by the ethical committee of our institution and all patients signed informed consent (patient population and controls).

### 2.2. Biochemical Measurements

Fasting blood samples for plasma aldosterone concentration (PAC) and plasma renin activity (PRA) were obtained in all patients. Patients were comfortably laying in a clinostatic position for at least half an hour. The assay for aldosterone (PAC) was performed with diagnostic kits (Aldosterone Mirya, Technogenetics, Sesto, Italy). Normal ranges were 10–150 pg/mL supine and 30–320 pg/mL upright on a normal Na diet; intraassay and interassay coefficients of variation were both less than 5.6%, and the cross-reactivity of the antibody for aldosterone for other adrenal steroids was less than 0.001%. PRA was measured by radioimmunoassay (RIA) using commercial kits (Ren CTK, Sorin Biomedical, Irvine, CA, USA). The normal range sitting at rest and on a normal sodium diet was 0.2–2.8 ng/mL per h; intraassay and interassay coefficients of variation were within 8% and 10%, respectively. A cutoff upright PAC/PRA ratio (ARR) of less than 30 in the presence of aldosterone greater than 15 ng/dl and suppressed PRA was used as a screening test to exclude primary aldosteronism.

Cardiac Studies

All patients were evaluated with 2D-echocardiography, cardiac magnetic resonance (CMR), and invasive cardiac studies including coronary angiography and left ventricular EMB to clarify the etiology of cardiomyopathy. Biopsy samples were processed for histology, electron microscopy, immunohistochemistry, and Western blot analysis of myocardial aldosterone receptor and aquaporin 1.

Cardiac Magnetic Resonance

In all patients, a standard CMR examination was performed with a 1.5T system (Magnetom Avanto, Siemens Medical Systems, Erlangen Germany) using a body and phased array coils. Scanning protocol consisted of the acquisition of TSE T2w-STIR, cine SSFP (steady-state free precession), and delayed enhancement (DE) sequences 12–18 min after gadolinium administration; on short-axis images, the left ventricle was completely encompassed from the base to the apex, acquiring a total of 10–12 images. T1 mapping imaging was also performed in all cases before and after contrast, using a modified look-locker (MOLLI) sequence acquired in short axis at basal, mid, and apical segments before and after contrast. All CMR studies were analyzed to consensus by two experienced observers blinded to the EMB results and using a workstation with dedicated cardiac software (cmr42© v.40, Circle Cardiovascular Imaging Inc., Calgary, AB, Canada).

Histology, electron microscopy and molecular studies

A total of 5 to 8 endomyocardial biopsy samples from each patient were cut and processed for routine histological analysis and for transmission electron microscopy (TEM). In those patients with inflammatory cardiomyopathy at histology, two frozen samples were processed for PCR for the most common cardiotropic viruses, including adenovirus, enterovirus, influenza A/B, parvovirus B19, cytomegalovirus, HHV6, HSV1, HSV2, EBV, and HCV. In addition, two myocardial fragments from each patient were processed for assessment of aldosterone receptor and aquaporin channels. For histological analysis, the endomyocardial samples were fixed in 10% buffered formalin and paraffin embedded. Five micron-thick sections were stained with hematoxylin & eosin, Masson trichrome, and Miller’s Elastic Van Gieson. For TEM, samples were fixed in 2% glutaraldehyde in a 0.1 M phosphate buffer at pH 7.3, post fixed in osmium tetroxide, and processed following a standard schedule for embedding in Epon resin. Ultrathin sections were stained with uranyl-acetate replacement stain (EMS #22405, from EMS, Hatfield, PA, USA) and lead hydroxide. A Jeol-1400-plusTEM was used for observation and photographic analysis. 

Assessment of myocardial aldosterone receptors and aquaporin channels

*Immunohistochemistry*

The expression of aldosterone receptor (AR) and aquaporins (AQP) was evaluated by immunoperoxidase using mineralocorticoid receptor monoclonal antibody (1:100, Enzo Life Sciences, Inc.Farmingdale, NY, USA) and Aquaporin 1 (AQP1) mouse monoclonal antibody (1:80, Santa Cruz Biotechnology, Inc., Dallas, TX, USA) as primary antibodies. Intensity of immunostaining was semi quantitatively evaluated as absent (grade 0), weak (grade 1), mild (grade 2), moderate (grade 3), or strong (grade 4). For each patient, the grading was calculated on 10 different histological sections, and the average value was computed.

*Protein isolation and western blot*

Heart tissue samples were treated as described [10]. The expression of myocardial AR and AQP1 was visualized by using mineralocorticoid receptor monoclonal antibody (1:500, Enzo Life Sciences, Inc., Farmingdale, NY, USA), anti-Aqp1 (1:100, Santa Cruz Biotechnology, Inc., Dallas, TX, USA). Anti-α-sarcomeric actin (1:500, Sigma-Aldrich, Saint Louis, MO, USA) antibody was used for normalization. Signal was visualized using a secondary horseradish peroxidase-labeled goat anti-mouse antibody (goat anti-mouse IgG-HRP 1:5000, Santa Cruz Biotechnology) and enhanced chemiluminescence (ECL Clarity Bio-Rad, Menlo Park, CA, USA). The purity as well as equal loading (40γ) of the protein was determined by Nanodrop One (Thermofisher, Waltham, MA, USA). Digital images of the resulting bands were quantified by the Image Lab software package (Bio-Rad Laboratories, Munchen, Germany) and expressed as arbitrary densitometric units.

Statistical analysis 

Normal distribution of variables was assessed with the Kolmogorov-Smirnov test. For continuous variables, descriptive statistics were provided (number of available observations, mean, median, standard deviation), while median (interquartile range) was used for non-normal data. Categorical data were described as number (percentage). Baseline demographic and clinical characteristics are presented in table format. The Student *t*-test, x^2^ test, and Fisher exact test were used for comparisons. Differences between variables with no normal distribution were tested with the Mann-Whitney U test. For all tests, a *p* value less than 0.05 was considered statistically significant.

Changes observed before and after immunosuppressive treatment were examined by paired t-test and McNemar test.

To normalize target protein expression, the band intensity of each sample was determined by densitometry with ImageJ software. Next, the intensity of the target protein was divided by the intensity of the loading control protein. This calculation adjusted the expression of the protein of interest to a common scale and reduced the impact of sample-to-sample variation. Relative target protein expression could then be compared across all lanes to assess changes in target protein expression across samples. The change of AR and AQ1 expression in human failing heart and after therapy was calculated using the Wilcoxon matched-pairs signed-rank test.

The statistical analysis was performed using SPSS version 25.0 for Windows (IBM Software, Inc., Armonk, NY, USA).

## 3. Results

Twenty-six patients were included in the study; 20 were males (77%), median age was 58.4 ± 11.4 years. Comparison of baseline biochemical, clinical, and endomyocardial biopsy data among patient population (Group A) and normal controls (Group B) are shown in Table 1.

Cardiac studies

**Figure 1:**
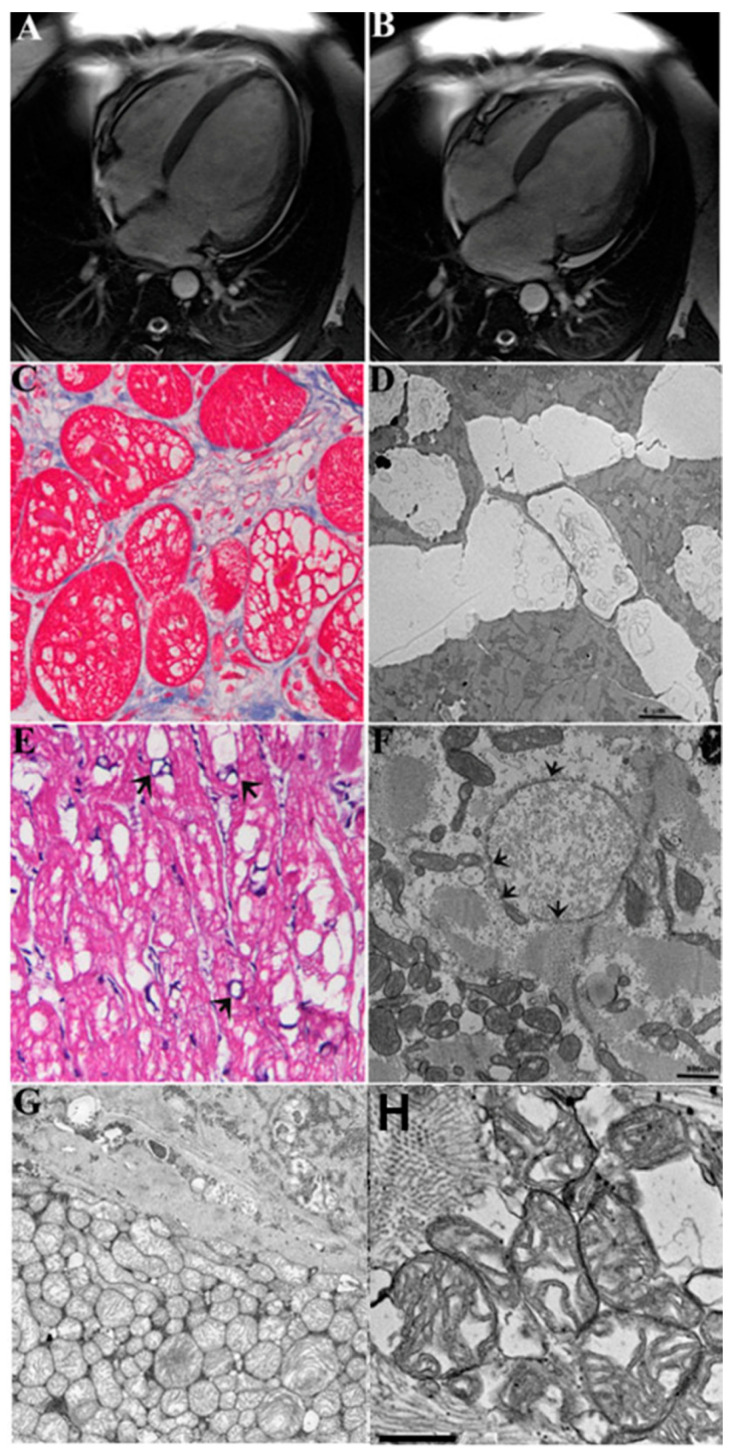
CMR, histologic, and ultrastructural changes of cardiomyocytes in a 45-year-old patient during heart failure caused by myocarditis. Panels (**A**,**B**): Cardiac magnetic resonance (CMR) showing bi-ventricular dilatation and severe dysfunction (LVEF 25%). Panels (**C**–**H**): LV endomyocardial biopsy showing vacuolar degeneration of myocytes (**C**) corresponding at electron microscopy to water accumulation in clear cisternae in the endoplasmic reticulum (**D**) (The bar represents 4 μm), nucleus (**E**,**F**), the bar represents 800 nm) and vesiculated mitochondria (**G**) ×3000 original magnification; (**H**)—The bar rep-resents 500 nm). In panel (**F**), remarkable nuclear edema causes chromatolysis and cell death; arrows indicate fragmentation of nuclear membrane. Further details of nuclear changes are better shown in Supplemental Figures S11 and S17.

Two-dimensional echocardiography showed a dilated left ventricle (LVEDD 61.5 ± 7.4 mm) with severely reduced ejection fraction (LVEF % 27.8 ± 5.8). Cardiac magnetic resonance (CMR) confirmed LV dilation (LVEDV 226.8 ± 69.6 mm) and dysfunction (LVEF % 26.3 ± 7.1) (Figure 1 panels A, B). Areas of late gadolinium enhancement were detectable in the interventricular septum and infero-posterior LV wall in patients with dilated CM, alcoholic CM, diabetic CM, and long-lasting inflammatory cardiomyopathy, suggesting increased myocardial fibrosis. In patients with active myocarditis, CMR showed areas of late gadolinium enhancement (subepicardial, mid-wall, or diffuse) corresponding to myocardial edema on T2-weighted images and increased native T1 values on mapping. Coronary network was normal in all patients.

Endomyocardial biopsy studies and ultrastructural findings

Histological diagnosis was virus-negative inflammatory cardiomyopathy in 15 patients (57.7%), idiopathic non-familiar dilated cardiomyopathy in 6 patients (23.1%), and in the remaining cases (19.2%), the etiology of heart failure was alcoholic cardiomyopathy (n = 2) and diabetic cardiomyopathy (*n* = 3) [11].

In all patient population, regardless of the etiology of heart failure, histology showed an increased volume of cardiomyocytes, containing large intracellular vacuoles (vacuolar or hydropic degeneration) and a typical cell swelling (or cloudy swelling), as described by Virchow [12] (Figure 1 panel C, Supplemental Figure S1, Supplemental Figure S2 Panel A, and Supplemental Figure S3).

At TEM, a number of subcellular changes were constantly present, all directly or indirectly related to the cell volume changes and water and ion accumulation. The Supplemental Table S1 summarizes these changes and their recovery after therapy.

Large vacuoles contained an electron-clear material (Figure 1 panel C), mostly corresponding to the enlarged cisternae of endo/sarcoplasmic reticulum, Golgi apparatus, and other subcellular compartments (Supplemental Figures S4–S17). Cytosol also appeared in electron-clear dilution with large regions of myofibrillolysis (Figure 1 panel D and Supplemental Figures S10 and S11). Noteworthy, in most of cardiomyocytes, mitochondria were uniformly swollen (Figure 1 panel F) with highly diluted/vesiculated matrix and disorganized cristae; in other cardiomyocytes the *size* varied greatly, suggesting an abnormal dynamic in the fusion/fission process. Moreover, the *distribution* of mitochondria along the cell was highly irregular, being mostly grouped in large clusters, in contrast with the columnar arrangement seen in a normal myocardium (Supplemental Figures S12–S17). Nuclear changes included swelling of nucleoplasma, chromatolysis, nucleolar disorganization, and nuclear membrane alterations (Figure 1 panel F; Supplemental Figures S11 and S17). The intercalated disk sometimes was focally disorganized, and lateral myocardiocyte junctions appeared mostly disorganized by the increased spaces among cells (extracellular edema, as seen in Supplemental Figures S4–S7).

Normal myocardium is represented in Supplemental Figures S19 and S20.

Importantly, in all these tissues, AR, ion channels and transporters, and aquaporins have been explored in many other papers [13,14,15,16,17,18,19,20], suggesting their involvement in ion/water compartment changes.

**Figure 2:**
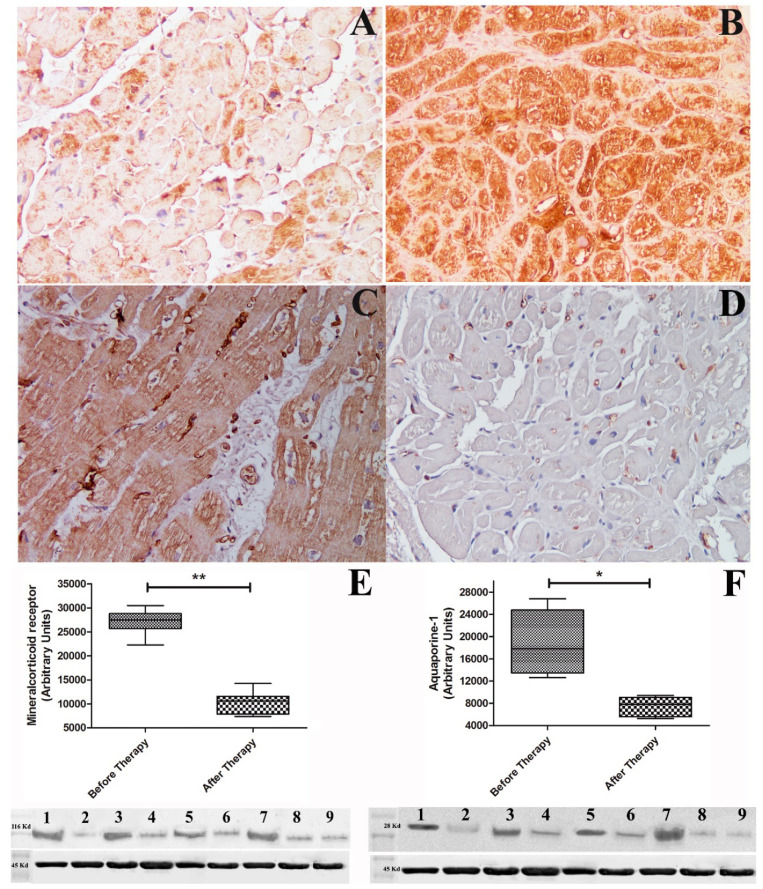
Immunostaining and Western blot quantification of aldosterone and aquaporin 1 receptor under heart failure and after cardiac recovery. Panels (**A**,**B**): panel (**A**) shows reduced immunostaining for aldosterone receptor that remarkably increases (**B**) with enhancement of cardiac contractility (LVEF from 22% to 55%). Panels (**C**,**D**): Increased immunostaining for aquaporin 1 in dysfunctioning heart (**C**) that disappears (**D**) after cardiac recovery (Figure magnification 200×). Panel (**E**): Western blot of aldosterone receptor (116,000 Da) showing 2.6 increase vs. control during heart failure, which returns to normal after recovery of cardiac function. Panel (**F**): Western blot of myocardial aquaporin 1 (28,000 Da) showing 2.7 fold increase vs. control normalizing after therapy. Alpha sarcomeric actin (45,000 Da) was used as loading control. Lanes 1, 3, 5, 7 = Western blot of patients during heart failure. Lanes 2, 4, 6, 8 = Western blot of patient after recovery. Lane 9 = control. * indicates *p* value < 0.05; ** indicates *p* value < 0.001.

Immuno-histochemistry for AR showed a lower grading in heart failure (Figure 2 panel A) because of aldosterone competitive binding [21]; conversely Western blot of aldosterone receptor showed 2.6-fold increase in protein expression (Figure 2 panel E). Immunostaining for AQP1 was elevated (Figure 2 panel C) as well as Western blot of myocardial AQP1 that showed 2.7-fold increase in dysfunctional heart (Figure 2 panel F).

Treatment and follow up

All patients were treated according to the current heart failure guidelines of the European Society of Cardiology with carvedilol, angiotensin-converting enzyme inhibitors (or angiotensin receptor blockers in patients unable to tolerate angiotensin-converting enzyme inhibitors), digitalis, diuretics in case of congestion, nitrates, mineralocorticoid/aldosterone receptor antagonists (spironolactone 25 mg daily), and if needed, antiarrhythmic drugs [22] In particular, all patients were treated with betablockers, 42% with ACE inhibitors, 23% with angiotensin receptor blockers, 35% with sacubitril/valsartan, 92% with diuretics, 19% with nitrates, 26% with digoxin, 31% with amiodarone; all patients were treated with aldosterone inhibitors. The fifteen patients with virus-negative inflammatory cardiomyopathy were treated with immunosuppressive therapy, including prednisone 1 mg/kg per day for 4 weeks, followed by 0.33 mg/kg per day for 5 months and azathioprine 2 mg/kg per day for 6 months according to the TIMIC (Immunosuppressive Therapy for Inflammatory Cardiomyopathy trial [9] in addition to standard therapy for heart failure. At the end of treatment, 8 of them accepted a control left ventricular EMB.

All patients were clinically followed up at 6 months with ECG, echocardiogram, and CMR. As a consequence of optimized heart failure treatment, all patients showed an improvement in clinical status and better exercise tolerance. No patient died. In particular, LVEF in patients with dilated CM, alcoholic CM, and diabetic CM raised from 23.4 ± 4.2% to 30.1 ± 5.9%. Better metabolic control in diabetic patients and withdrawal from alcohol in diabetic patients contributed to clinical recovery.

**Figure 3:**
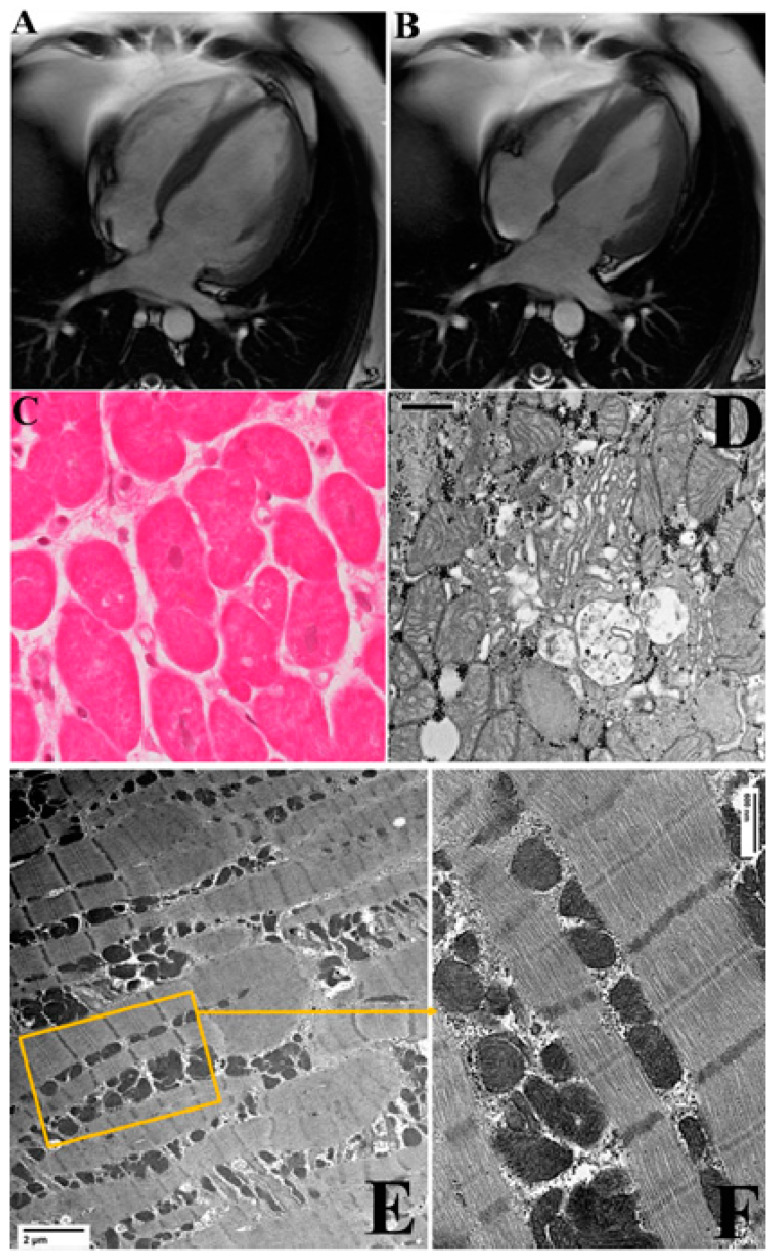
Clinical, histologic, and ultrastructural changes of cardiomyocytes in the 45-year-old patient with myocarditis presented in Figure 1, after cardiac recovery by immunosuppressive therapy. Panels (**A**,**B**): Control CMR documenting recovery of cardiac dimension and function with LVEF rising to 56%. Panel (**C**): Optical microscopy shows reduction in cardiomyocytes dimension with disappearance of vacuoles and decrease in extracellular spaces following cardiac recovery. Panel (**D**): Hypertrophic/hyperfunctioning Golgi apparatus with enlarged vesicles for water/ion extrusion (The bar represents 600 nm). Panels (**E**) Recovery of ultrastructure with normal sarcomere and mitochondrial organization. The bars represent 2 μm. Panel (**F**): detail shows energized mitochondria in columnar distribution among normal well-organized sarcomeres. The bars represent 600 nm. and 600 nm in (**F**).

Patients with virus-negative inflammatory cardiomyopathy showed a more pronounced clinical benefit after the immunosuppressive therapy, with an increase in LVEF (Table 2). Cardiac dimensions reduced at CMR (LVEDV baseline: 270.8 ± 51.9 mm; LVEDV at 6 month-follow up: 205.8 mm ± 42.7 mm) and LVEF percentage rose from 23.3 ± 5.2% to 52.6 ± 5.9% (Figure 3 panels A, B). Moreover, myocardial edema was no longer detectable at CMR, and late gadolinium-enhanced images were attenuated. In patients who accepted a control biopsy, histology showed healed myocarditis with reduced volume of cardiomyocytes, disappearance of large vacuoles, and recovery from most alteration of subcellular compartments described above (Figure 3 panels C, D).

A detailed description of the recovery of each cell compartment is shown in the Supplemental Table S1. In particular, electron micrographs show an advanced reorganization of the swollen altered cardiomyocytes, with a general recovery from swelling and vacuolar degeneration, an almost normal distribution among myofibrils of mitochondria with an orthodox/dense energized conformation, recovering from swelling and vesiculation. Of note, Golgi apparatus hypertrophy in all three cis, medial, and trans components suggests a role in the vesicular extrusion of water and ions [13,14]. Vacuoles of autophagy and extrusion of exosomes are also frequently seen in recovering samples after therapy (Supplemental Figure S2); both autophagy and large vesicle extracellular release could be other mechanisms for recovering from changes induced by hyperaldosteronism.

Additionally, aldosterone receptor and aquaporin channels that were both equally overexpressed in heart failure were normalized after cardiac recovery (Figure 2 panels B, D, F).

## 4. Discussion

Abnormal aldosterone signaling has been recognized to adversely affect the cardiovascular system. On the other hand, contrast of aldosterone activity in the failing heart by aldosterone inhibitors has been found clinically beneficial and to be followed by a reduction in left ventricular hypertrophy and decrease in mortality and cardiovascular-related hospitalizations compared with placebo [1,2,3,4]. On the base of clinical studies, aldosterone inhibitors have been recommended by the current guidelines in the pharmacologic treatment of heart failure [5,6]. 

Nevertheless, the cellular and morpho-molecular basis of aldosterone damage and of its recovery is still unknown. 

In the present report, analyzing pre- and post-recovery endomyocardial biopsy samples, it is documented that, in the failing heart, increased plasma aldosterone activity is associated with overexpression of myocardial aldosterone receptor and aquaporin-1. In particular, in Western blot analysis, both figures increased by more than 100%.

Role of aldosterone receptor 

AR dimer is responsible for a complex gene expression, including ion transporters, co-transporters, and ion channels, especially some Na^+^ channels, allowing sodium entry into the cell [18,23]. Therefore, increased AR signaling may alter ion distribution between cardiomyocytes and extracellular spaces and in the cytoplasm among different subcellular compartments. 

A transcriptional loop also activates the synthesis of new AR, determining an increased expression of AR according to Western blot analysis (Figure 2 Panel E).

Altering the ionic content of subcellular compartments and changing their osmolarity, the AVP2 (arginine/vasopressin receptor 2) is activated, producing AQP1 expression and increasing water movements along the osmotic gradient [13].

Role of Aquaporin-1 

In the myocardium are present three AQPs: AQP1 is the most expressed, and AQ3 and AQP7 are expressed to a lesser extent [13,24]. AQP1 is more abundant in the ventricles and less in the atria and in the vessel wall (endothelial cells); its subcellular localization in biological membranes may vary with trafficking among cell membranes and in relation to the function required (as it has been shown in the formation of vacuoles/vesicles in the hepatocytes [13]).

Role of water and ion movements in damage production and its reversal

In the present study, overactivation and overexpression of AR is hypothesized to cause transcription of Na^+^ channels (and potential-activated Cl-channels), producing an intracellular Na^+^ (and anions) inflow with increased cell osmolarity that activates AQ1 channels with consequent water mobilization (Supplemental Figure S18). Water entrance into cardiac cells can be massive, requiring its compartmentalization in vesicles, but it is also retained in the cytosol and particularly into the organelles, including the endoplasmic reticulum (Figure 1 panel D), mitochondria (Figure 1 panel G), and Golgi apparatus (Supplemental Figures S12, S15 and S16). The cell nucleus appears affected as well by edema that may be severe enough to induce focal chromatolysis (Figure 1 panel E). Accordingly, several previous papers [13,14,17] have demonstrated that vacuoles and cytosol swelling with electron-clear material are produced by massive movements of ions and water accumulating both in the cytosol (cloudy swelling) and/or segregated into vacuoles (vacuolar degeneration), strictly associated with alterations of cell volume control mechanisms—not in the myocardium, but in several other tissues, such as in kidney [17], liver [13,14,15], smooth muscle [18,19,20], and lung [25].

Moreover, there is evidence in the literature that aldosterone receptor is up-regulated in the failing heart [26], and its overexpression can trigger cardiac arrhythmias as a consequence of prolonged ventricular repolarization [27]. Therefore, treatment with aldosterone inhibitors may have beneficial effects on cardiac function.

Possible mechanisms involved in the ultrastructural recovery

Although it is clear that after normalization of aldosterone signaling, the extrusion of ion and water is a relevant mechanism involved in the ultrastructural recovery of damaged cardiomyocytes, ultrastructural evidence suggests that other mechanisms may be involved in the normalization of myocytes architecture and function. The Supplemental Table S1 summarizes the pathways that are active during the ultrastructural recovery, being directly or indirectly associated with cell volume normalization. In particular, the recovery of osmolarity, the normalization of AQP, and the restoration of mitochondrial coupling (transition from swollen to orthodox/energized conformation) are directly related to the extrusion of water and ion [13,16,19]. Activation of autophagocytosis and extrusion of undigested/unwanted material may be secondary to the restoration of energy levels obtained in parallel with mitochondrial recovery. Finally, mitochondrial dynamics (distribution and fission/fusion) and sarcomere reorganization can also be related to the cytoskeletal restoration secondary to cell volume recovery, ion homeostasis, and energy production [13].

In addition, morpho-molecular changes observed in our patient population with heart failure are similar to those described in cardiomyopathy associated with primary aldosteronism [7].

Pathophysiologic consequences of electrolytes and water cell inflow appear to be cardiomyocyte swelling and likely functional impairment. In particular, it is believed that intracellular electrolytes (including cytosolic Ca + + homeostasis) and water overloading might negatively influence myocyte relaxation and contraction contributing to an impairment of diastolic and systolic cardiac function.

In support of this interpretation, endomyocardial biopsies obtained after recovery of cardiac contractility show reduction in size as well as normalization of cardiomyocyte composition in parallel with the down-regulation of cell receptors. 

Noteworthy, myocardial edema was at least in part independent of the cause of heart failure. Indeed, patients with myocarditis as well as subjects with various types of dilated cardiomyopathy manifested the same histologic and ultrastructural findings correlated with cardiomyocyte water accumulation. This also suggests that the effect of AR signaling activation may also affect mesenchymal components of the myocardium, including vessels and fibroblasts, the swelling of which may contribute to the accumulation of water and ions in the extracellular space (edema). The Supplemental Figure S18 shows a hypothetical sequence triggered by AR signaling, leading to ion/water homeostasis disruption, ultrastructural changes, and cardiomyocyte damages.

Finally, our data confirm the negative impact of aldosterone pathway activation in the failing heart and the potential benefit from administration of aldosterone inhibitors.

Limitations of the Study 

Our study, through sequential analysis of endomyocardial biopsies suggests that heart failure, independent of etiology, is associated with overexpression of AR and AQP1. A major limitation of the present study is that there is no definite proof of a cause-and-effect relationship between the molecular and the histological changes. Nevertheless, a previous report on endomyocardial findings associated with primary aldosteronism cardiomyopathy [7] showed similar aspects supporting these observations. In addition, considering the transcriptional profile of AR dimer in different tissues, including the myocardium, it is very likely that ion channels, pumps, and transports—and, indirectly, AQPs—are responsible for electron-clear large vacuoles whose disappearance is sensitive to AR signaling inhibition/normalization.

To avoid a confounding effect on myocardial expression of AR, patient enrolment was limited to those subjects not taking aldosterone inhibitors. They started spironolactone only after histological and ultrastructural diagnosis and study.

While the present study shows for the first time the ultrastructural myocardial changes associated with secondary aldosteronism, it is unable to quantify its impact in terms of impairment of cardiomyocyte relaxation and contraction, as well as its potential role in the generation of cardiac electrical instability.

Further unanswered points are the quantification of structural and functional recovery inducible by aldosterone-inhibitors and the determination of their most appropriate dosage.

Finally, novel experimental studies in human myocardial organoid or in animal models [28] are necessary and mechanistically informative, analyzing cardiomyocyte structural and receptor changes following aldosterone-inhibitors treatment.

## 5. Conclusions

Human heart failure is associated with a reversible up-regulation of myocardial AR and AQP1. These molecular changes are paralleled by intracellular ion/water overloading, (cell swelling and large vacuole formation and a number of changes in other subcellular components) and by a likely compromise of contractile function. For a cause/effect demonstration and a quantification of morpho-molecular effects of aldosterone inhibitors and their ideal dosage, further studies are needed.

## Figures and Tables

**Table 1:** Comparison of baseline biochemical, clinical, and endomyocardial biopsy data among patients with human heart failure (Group A) and normal controls (Group B).

	Group A N = 26	Group B N = 26	*p* Value *
Age	58.4 ± 11.4	57.5 ± 11.5	0.818
Sex (M/F) †	20M, 6F	17M, 9F	0.541
BMI (kg/m^2^) ‡	26.4 ± 3.6	23.1 ± 3.2	0.0759
SBP (mmHg) §	114.6 ± 15.2	111.9 ± 9.1	0.9249
DBP (mmHg) ||	68.8 ± 9.5	66.3 ± 6.2	0.4204
HR (bpm) #	79.1 ± 12.8	74.5 ± 5.5	0.4854
Blood values			
Creatinine (mg/dL)	1.3 ± 0	0.9 ± 0.2	0.01
Na (mmEq/L)	139.1 ± 3.1	137.7 ± 2.6	0.05
K(mmEq/L)	4.0 ± 0.4	3.9 ± 0.2	0.3032
Ca (mmEq/L)	10.0 ± 0.4	9.2 ± 0.4	0.2614
PAC (pg/mL) **	185.4 ± 68.7	61.6 ± 16.1	0.05
PRA (pg/mL) ***	18.8 ± 7.8	4.6 ± 1.8	0.0001
PAC/PRA ratio	8.6 ± 6.4	13.1 ± 3.4	0.01
2d-Echocardiography			
MWT (mm) ††	11.0 ± 2.6	9.6 ± 0.9	0.01
LVEDD (mm) ‡‡	61.5 ± 7.4	44.4 ± 4.5	0.0001
LVEF% §§	27.8 ± 5.8	59.7 ± 4.2	0.0001
Cardiac Magnetic Resonance Imaging			
MWT (mm) ††	11.3 ± 2.4	9.8 ± 0.9	0.01
LVEDV (ml) |||	226.8 ± 69.6	82.6 ± 4.0	0.0001
LVESV (ml) ##	169.5 ± 62.9	30.1± 4.2	0.0001
LVEF (%) §§	26.3 ± 7.1	61.6 ± 16.1	0.0001
LV mass (g)	187.5 ± 70.1	87.8 ± 19.2	0.0001
Endomyocardial biopsy studies			
Cardiomyocyte diameter (µm)	34.5 ± 3.6	15.3 ± 2.5	0.0001
Fibrosis (%)	13.6 ± 1.8	1.5 ± 0.46	0.0001
Aldosterone receptor grade (0/4)	1.6 ± 0.8	2.9 ± 0.7	0.0001
AQP1 **** grade (0/4)	3.3 ± 0.5	1.4 ± 0.3	0.0001

* *p* values refer to comparison between the two groups. *p* value < 0.05 was considered statistically significant. Quantitative measurements are expressed as mean ± SD. † M = male, F = female, ‡ BMI = body mass index, § SBP = systolic blood pressure, || DBP = diastolic blood pressure, # HR = heart rate, ** PAC = plasma aldosterone concentrations, *** PRA = plasma renin activity, PAC/PRA ratio = plasma aldosterone concentration/plasma renin activity, †† MWT = maximal wall thickness, ‡‡ LVEDD = left ventricular end-diastolic diameter, §§ LVEF = left ventricular ejection fraction, ||| LVEDV = left ventricular end-diastolic volume, ## LVESV = left ventricular end-systolic volume, **** AQP1 = aquaporin 1.

**Table 2:** Comparison of biochemical, clinical, and endomyocardial biopsy data in 8 patients with heart failure: baseline and at 6-month follow up.

	Baseline N = 8	At 6-Month FU N = 8	*p* Value *
Age	55.5 ± 15.1	55.5 ± 15.1	
Sex (M/F) †	6M, 2F	6M, 2F	
BMI (kg/m^2^) ‡	27.6 ± 4.3	26.0 ± 3.5	0.4
SBP (mmHg) §	112.5 ± 11.6	114.3 ± 5.6	0.8294
DBP (mmHg) ||	67.5 ± 7.1	71.6 ± 3.4	0.1376
HR (bpm) #	81 ± 9.9	70.0 ± 6.9	0.05
Blood values			
Creatinine (mg/dL)	1.1 ± 0.1	0.8 ± 0.2	0.05
Na (mmEq/L)	139.5 ± 3.0	139.6 ± 1.8	0.9577
K(mmEq/L)	4.1 ± 0.25	4.0 ± 0.1	0.7080
Ca (mmEq/L)	9.2 ± 0.3	9.2 ± 0.3	0.9575
PAC (pg/mL) **	92.6 ± 65.4	57.5 ± 33.9	0.0830
PRA (pg/mL) ***	14.8 ± 12.8	10.0 ± 5.5	0.6454
PAC/PRA ratio	16.1 ± 18.1	5.6 ± 1.5	0.0582
2d-Echocardiography			
MWT (mm) ††	10.5 ± 2	9.7 ± 1.2	0.3506
LVEDD (mm) ‡‡	61.5 ± 7.4	54.8 ± 3.9	0.6731
LVEF% §§	26.2 ± 3.7	51.6 ± 5.8	0.0001
Cardiac Magnetic Resonance Imaging			
MWT (mm) ††	11.0 ± 2.7	10.1 ± 1.1	0.3601
LVEDV (ml) |||	270.8 ± 59.7	205.8 ± 42.7	0.05
LVESV (ml) ##	207.5 ± 51.9	97.3± 13.7	0.0001
LVEF (%) §§	23.3 ± 5.2	52.6 ± 5.9	0.0001
LV mass (g)	160.7 ± 46.6	139.2 ± 37.4	0.3823
Endomyocardial biopsy studies			
Cardiomyocyte diameter (µm)	32.6± 1.9	23.5 ± 2.6	0.0001
Fibrosis (%)	13.6 ± 1.8	13.6 ± 1.8	-
Aldosterone receptor grade (0/4)	1.5 ± 0.4	3 ± 0.5	0.0001
AQP1 grade (0/4)	3.4 ± 0.3	1.3 ± 0.4	0.0001
Aldosterone receptor Western blot (arbitrary units)	27,085 ± 2587	10,134 ± 2346	0.01
AQP1 **** Western blot (arbitrary units)	18,456 ± 5674	7537 ± 1667	0.05

* *p* values refer to comparison between two groups. *p* value < 0.05 was considered statistically significant. Quantitative measurements are expressed as mean ± SD. † M = male, F = female, ‡ BMI = body mass index, § SBP = systolic blood pressure, || DBP = diastolic blood pressure, # HR = heart rate, ** PAC = plasma aldosterone concentrations, *** PRA = plasma renin activity, PAC/PRA ratio = plasma aldosterone concentration/plasma renin activity, †† MWT = maximal wall thickness, ‡‡ LVEDD = left ventricular end-diastolic diameter, §§ LVEF = left ventricular ejection fraction, ||| LVEDV = left ventricular end-diastolic volume, ## LVESV = left ventricular end-systolic volume, **** AQP1 = aquaporin 1.

## Supplementary Materials

The following are available online at https://www.mdpi.com/article/10.3390/jcm10214854/s1, Figure S1: Baseline left ventricular endomiocardial biopsy, Figure S2: Transmission electron microscopy showing vesiculation before and its recovery after therapy, Figure S3: Baseline left ventricular endomyocardial biopsy showing hypertrophy, large/small vesicles, increased extracellular space, fibrosis, Figure S4: Baseline transmission electron microscopy showing hypertrophy, large vesicles, increased extracellular space (plastic section, Azur II stained), Figure S5: Baseline transmission electron microscopy showing vacuolar degeneration: large intracellular vesicles with clear content, Figure S6: Baseline transmission electron microscopy showing vacuolar degeneration: large intracellular vesicles with clear content enlarged extracellular space with clear content (extracellular edema), Figure S7: Baseline transmission electron microscopy showing vacuolar degeneration: large intracellular vesicles (IV) with clear content and extracellular edema (large amount of extracellular fluids in extracellular spaces [ES] and vessel lumen [VL]), Figure S8: Baseline transmission electron microscopy showing enlarged vessel lumen (VL) with osmiophilic myelin-like bodies (OMB), likely degenerating lipid membranes (A). Extracellular space also containing OMB (B), Figure S9: Baseline transmission electron microscopy showing enlarged vessel lumen (VL) (A) with electron clear material and/or osmiophilic myelin-like bodies (OMB), likely degenerating lipid membranes (B), Figure S10: Baseline transmission electron microscopy showing myofibrillolysis and chromatolysis with nuclear membrane damage, Figure S11: Baseline transmission electron microscopy showing nuclear changes (chromatin fragmentation, abnormal nucleolonema) and cytoplasm changes (areas of myofibrillolysis, abnormal sarcomeres and accumulation of lipofuscins), Figure S12: Baseline transmission electron microscopy showing alterations of mitochondrial dynamics/conformation and small vesicles dilatation (sarcoplasmic reticulum, Golgi vesicles, lysosomes, mitochondria), Figure S13: Higher magnification of A and B electron panels of the Figure S12, Figure S14: Baseline transmission electron microscopy showing alterations of mitochondria, Figure S15: Baseline transmission electron microscopy showing further details of dilated vesicles likely derived from sarcoplasmic reticulum, Golgi apparatus, lysosomes and other organelles, Figure S16: Baseline transmission electron microscopy showing detail of highly swollen mitochondria and other organelles, Figure S17: Perinuclear region with small vesicles to that observed in the Figure S16, Figure S18: An hypothetical description of the sequence triggered by aldosterone signaling leading to ion/water homeostasis disruption and to the myocardiocyte damage, Figure S19: Normal myocardium from EMB sample at histology, Figure S20: Normal myocardium from a EMB sample at transmission electron microscopy, Table S1: Transmission electron microscopy features before and after therapy in 8 patients with heart failure baseline and at 6-month follow up.

## Abbreviations

LVEF	left ventricular ejection fraction
EMB	endomyocardial biopsy
AD	aldosterone
PAC	plasma aldosterone concentration
PRA	renin activity
ICM	inflammatory cardiomyopathy
AR	aldosterone receptor
AQ1	aquaporin-1
