# Supplementary material for: Myocardial Aldosterone Receptor and Aquaporin 1 Up-Regulation Is Associated with Cardiomyocyte Remodeling in Human Heart Failure
Source: J Clin Med. 2021 Oct 22;10(21):4854. doi: 10.3390/jcm10214854 (PMC8585058; doi:10.3390/jcm10214854)
Supplement: Supplementary file 1 [file jcm-10-04854-s001.zip › jcm-1375629-supplementary materials.pdf]

Supplemental material

Supplemental Table S1. Transmission electron microscopy features before and after therapy in 8 patients with heart failure baseline and at 6-month follow up.

Features	Baseline	At 6-month follow up
Myocardiocyte axis	Increased	Decreased/normal
General swelling	evident	Recovered
Presence of large vesicles	Frequent	Absent
Sarcoplasmic reticulum	Dilated	Normal/partially recovered
Golgi cysternae	Irregularly dilated	Dilated (increased function)
Mitochondria	Swollen/vesculated	Orthodox/condensed/energized
Mitochondria dynamics	Abnormal fission/fusion	Apparently normal
Mitochondria distribution	Grouping	Mostly columnar
Intercalated disc	Occasionally disrupted	Apparently recovered
Lateral junctions	Disrupted	Recovered
Sarcomeres	Myofibrillolyisis	Recovered
Extracellular space	Dilated/highly enlarged (extracellular oedema)	Normal/partially recovered

Supplemental Figures showing baseline transmission electron microscopy findings

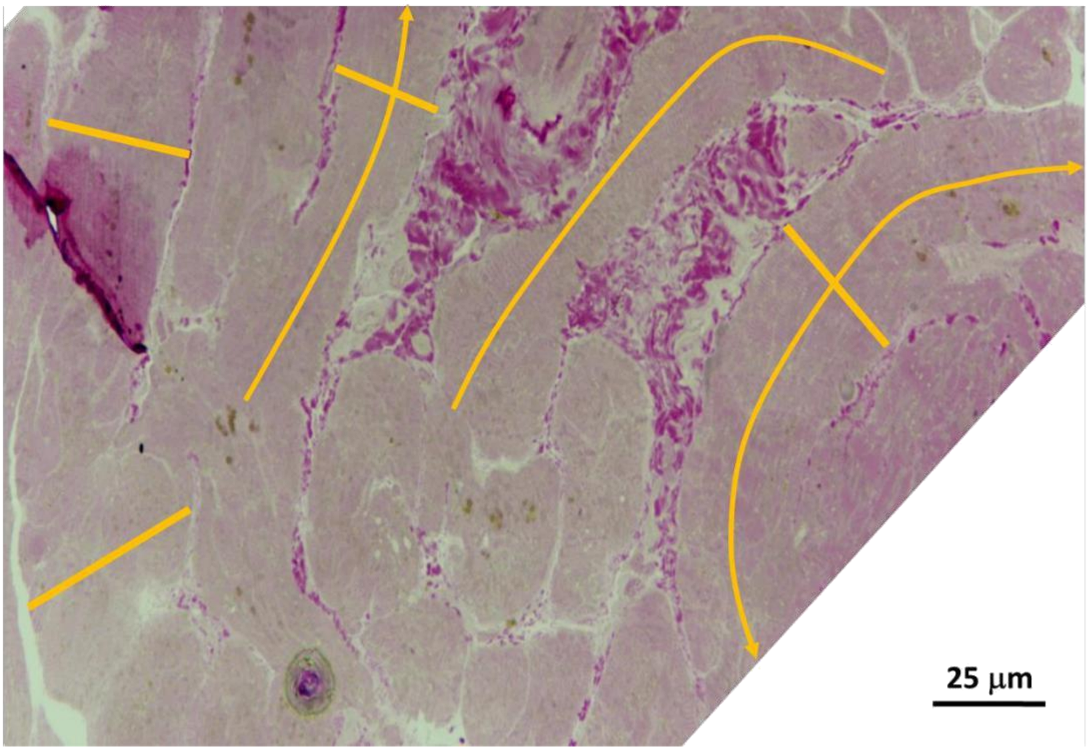

Supplemental Figure S1. Baseline left ventricular endomiocardial biopsy showing increased myocardiocyte axis and focal fibrosis

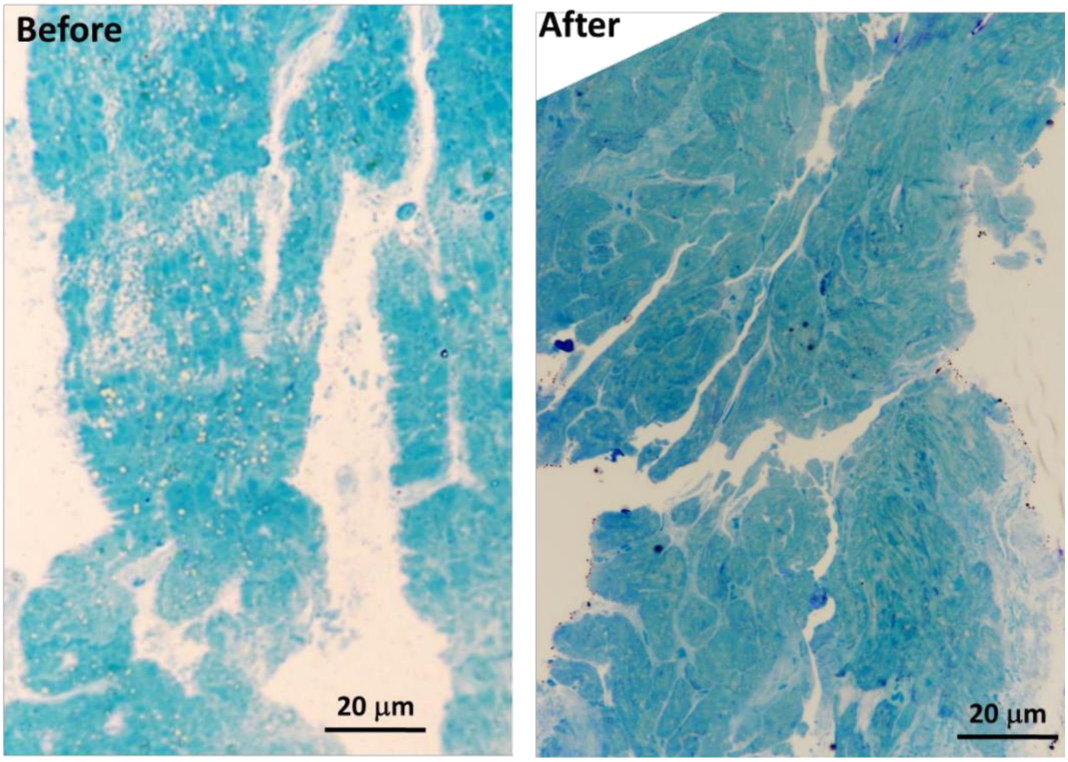

Supplemental Figure S2. Transmission electron microscopy showing vesiculation before and its recovery after therapy

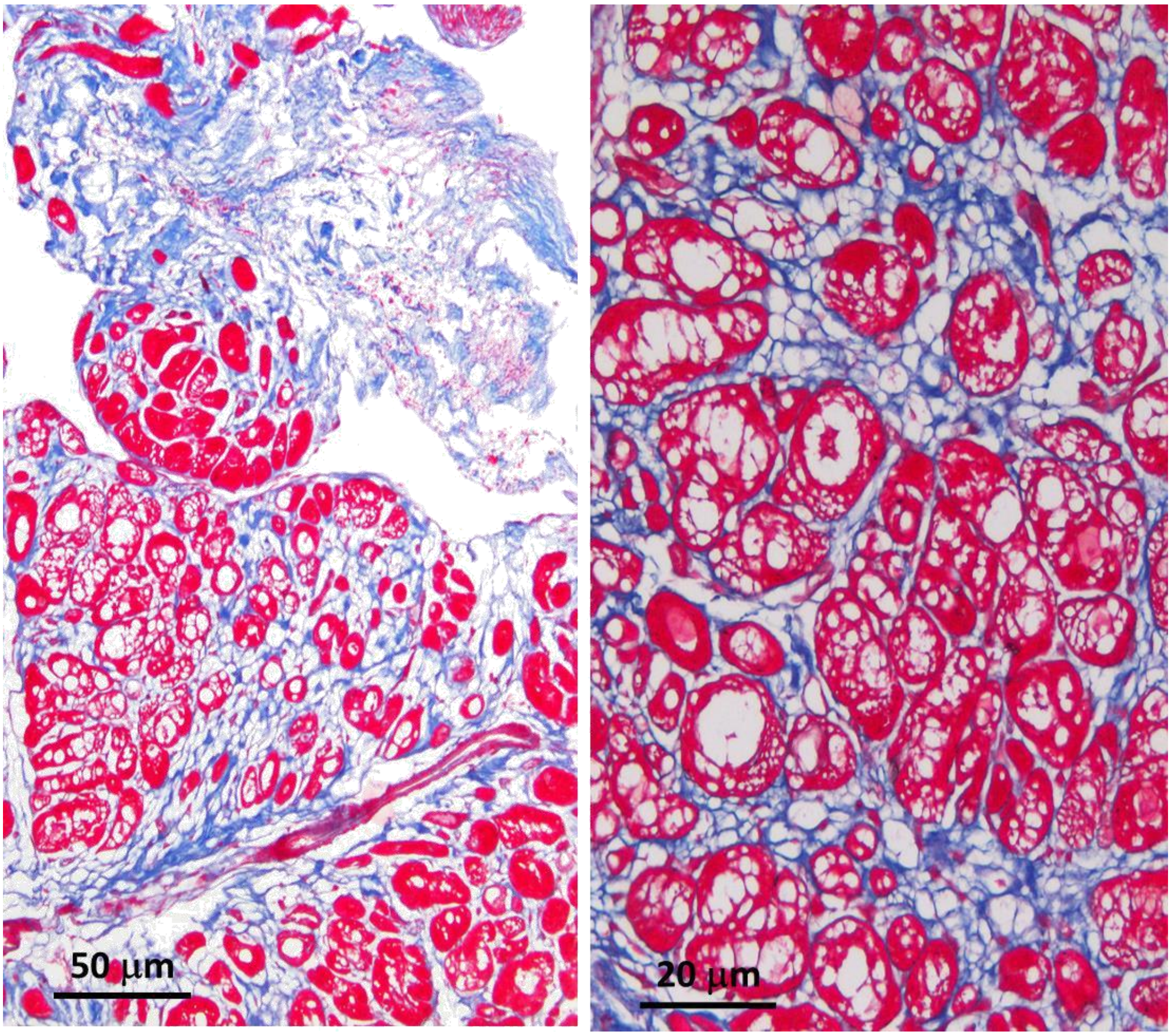

Supplemental Figure S3. Baseline left ventricular endomyocardial biopsy showing hypertrophy, large/small vesicles, increased extracellular space, fibrosis.

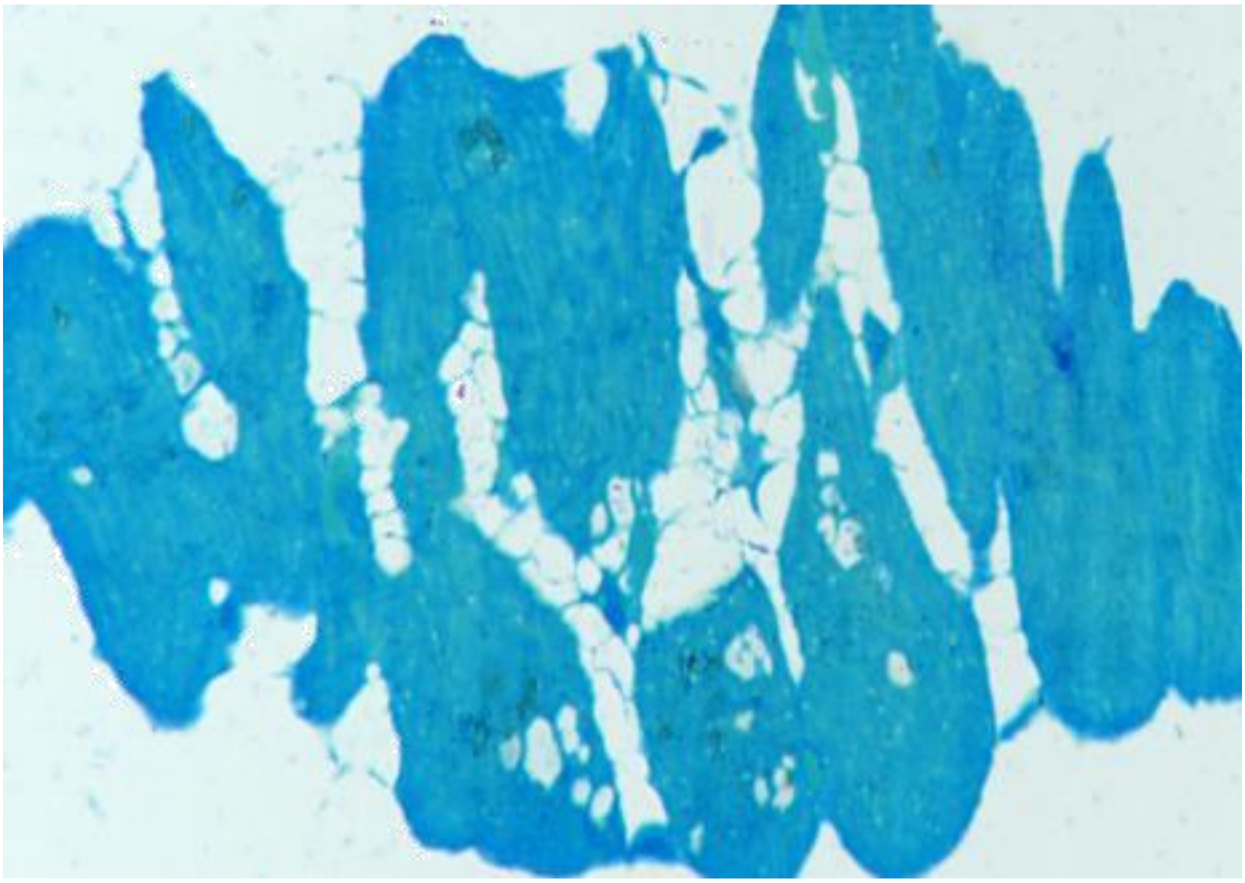

Supplemental Figure S4. Baseline transmission electron microscopy showing hypertrophy, large vesicles, increased extracellular space (plastic section, Azur II stained).

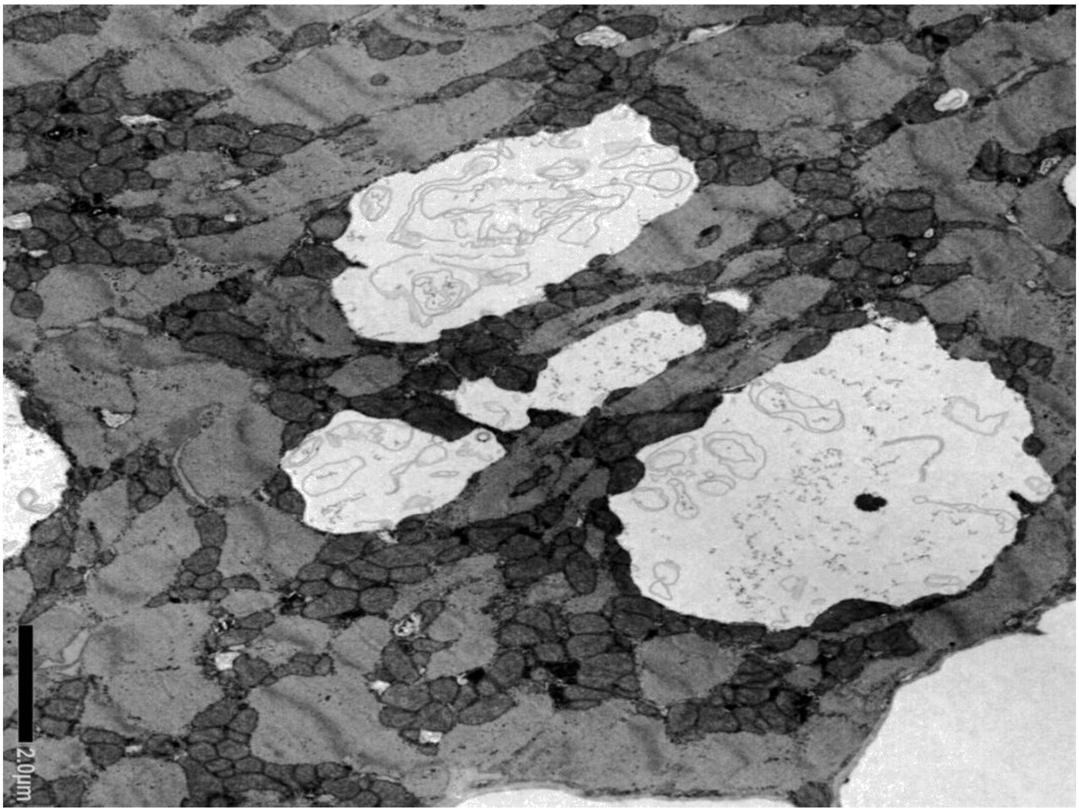

Supplemental Figure S5. Baseline transmission electron microscopy showing vacuolar degeneration: large intracellular vesicles with clear content.

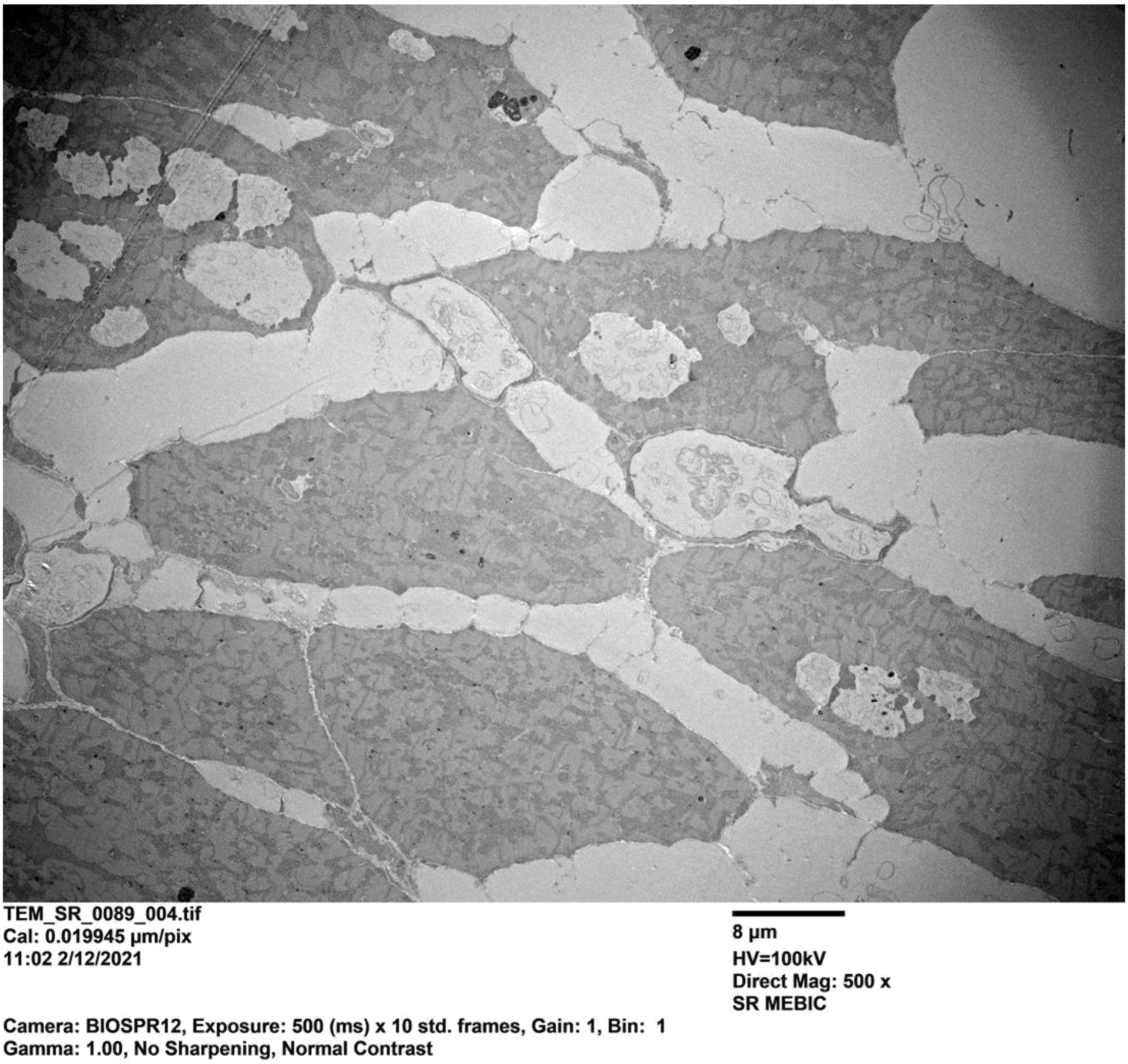

Supplemental Figure S6. Baseline transmission electron microscopy showing vacuolar degeneration: large intracellular vesicles with clear content enlarged extracellular space with clear content (extracellular edema).

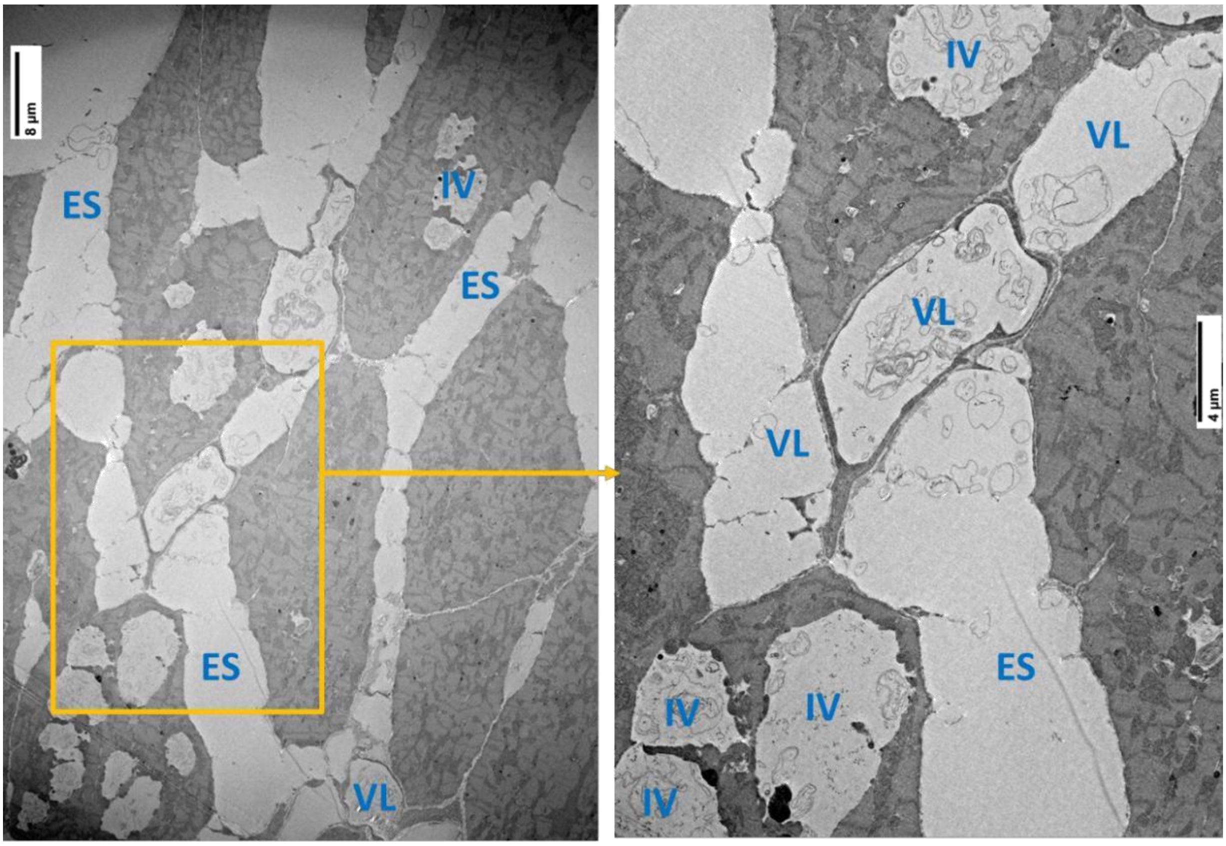

Supplemental Figure S7. Baseline transmission electron microscopy showing vacuolar degeneration: large intracellular vesicles (IV) with clear content and extracellular edema (large amount of extracellular fluids in extracellular spaces [ES] and vessel lumen [VL]).

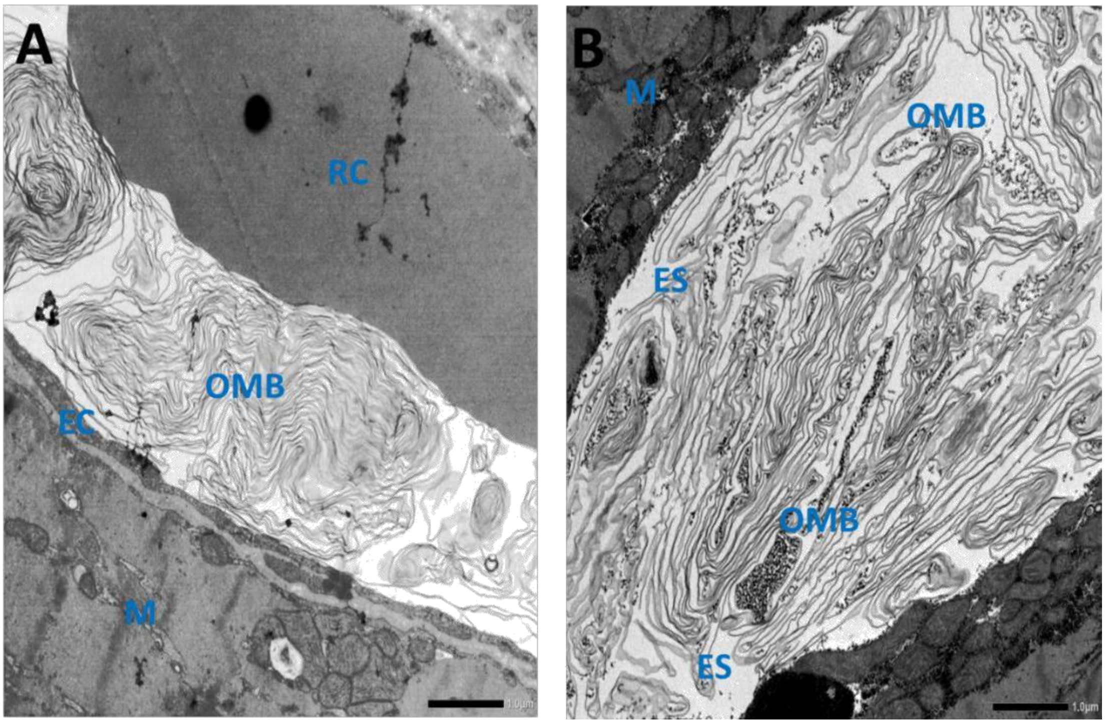

Supplemental Figure S8. Baseline transmission electron microscopy showing enlarged vessel lumen (VL) with osmiophilic myelin-like bodies (OMB), likely degenerating lipid membranes (A). Extracellular space also containing OMB (B).

EC = endotheliall cells; RC = Red Cell; ES = Extracellular Space

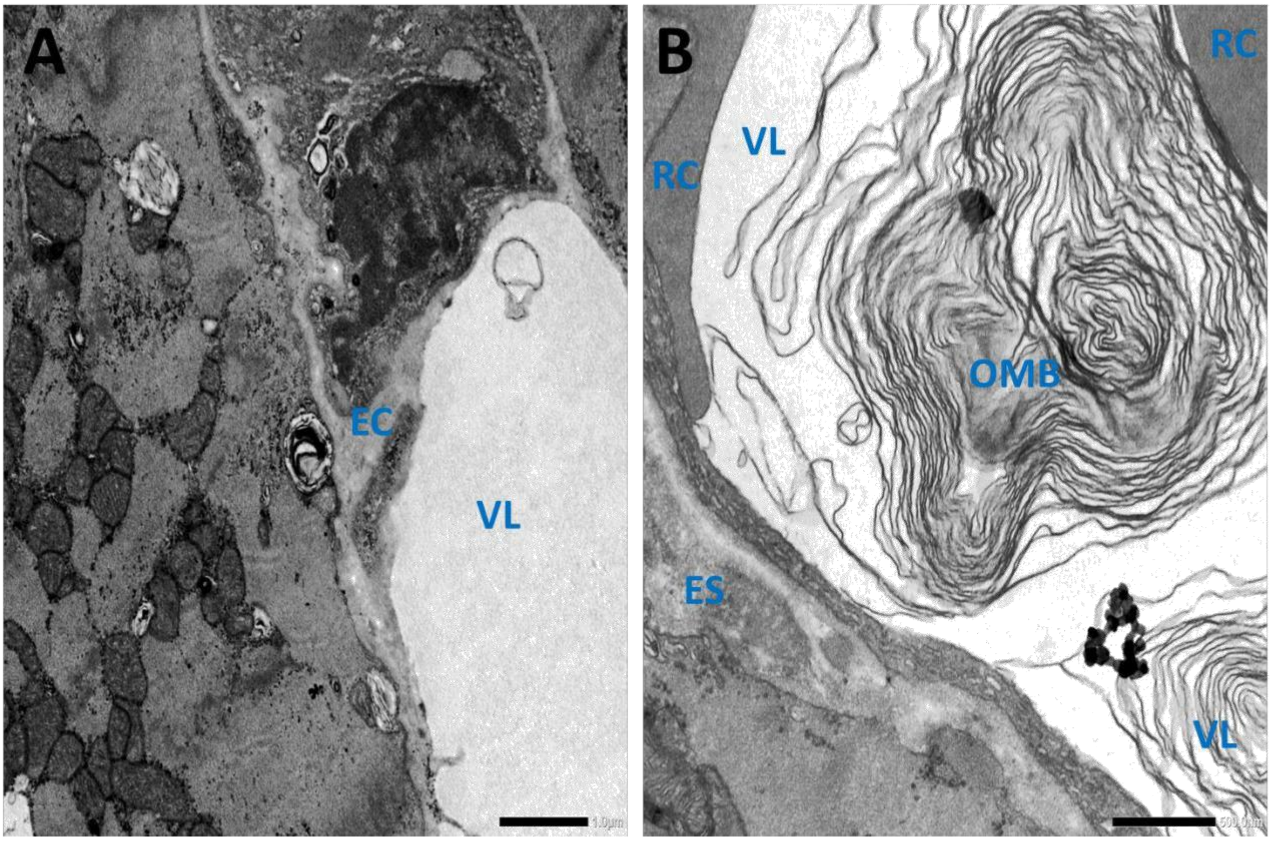

Supplemental Figure S9. Baseline transmission electron microscopy showing enlarged vessel lumen (VL) (A) with electron clear material and/or osmiophilic myelin-like bodies (OMB), likely degenerating lipid membranes (B).

EC = endotheliall cells; RC = Red Cell; ES = Extracellular Space

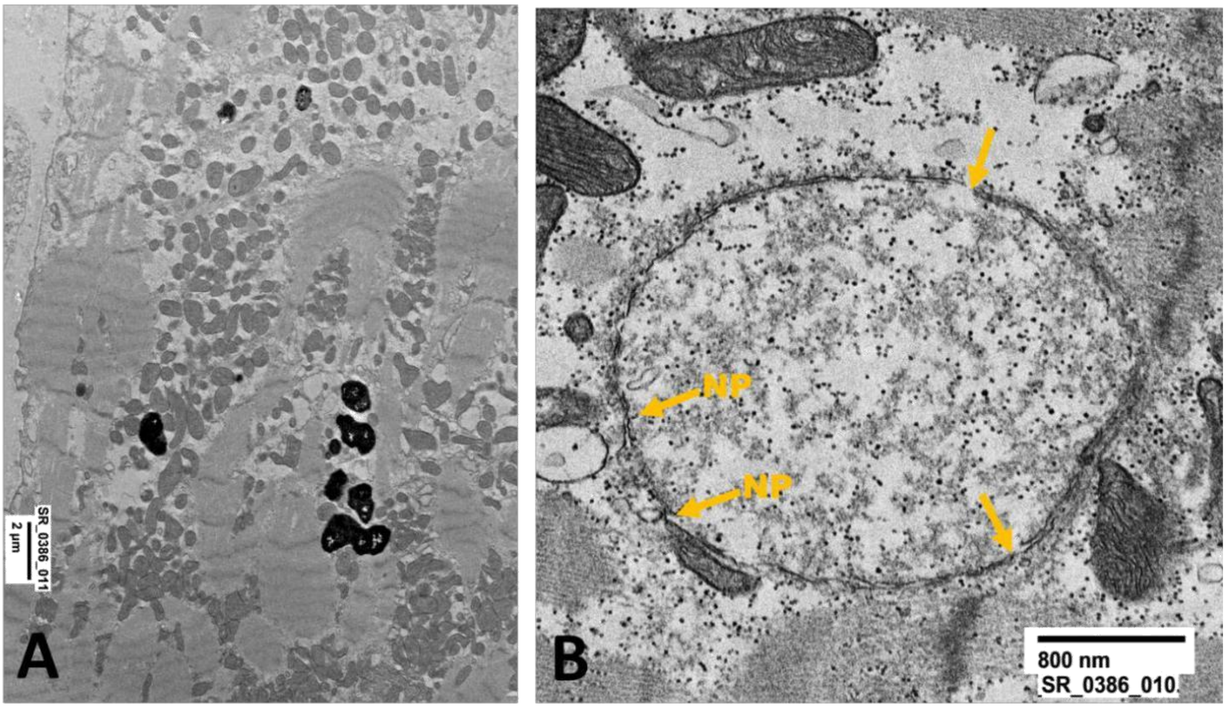

Supplemental Figure S10. Baseline transmission electron microscopy showing myofibrillolysis and chromatolysis with nuclear membrane damage.

A: Large areas of myofibrillolysis and sarcomere disorganization. Dense lipofiscin.

B: Nuclear damages: chromatolysis, nucleolar disorganization, nuclear membrane damages. NP = nuclear pore; Arrows = membrane fragmentation.

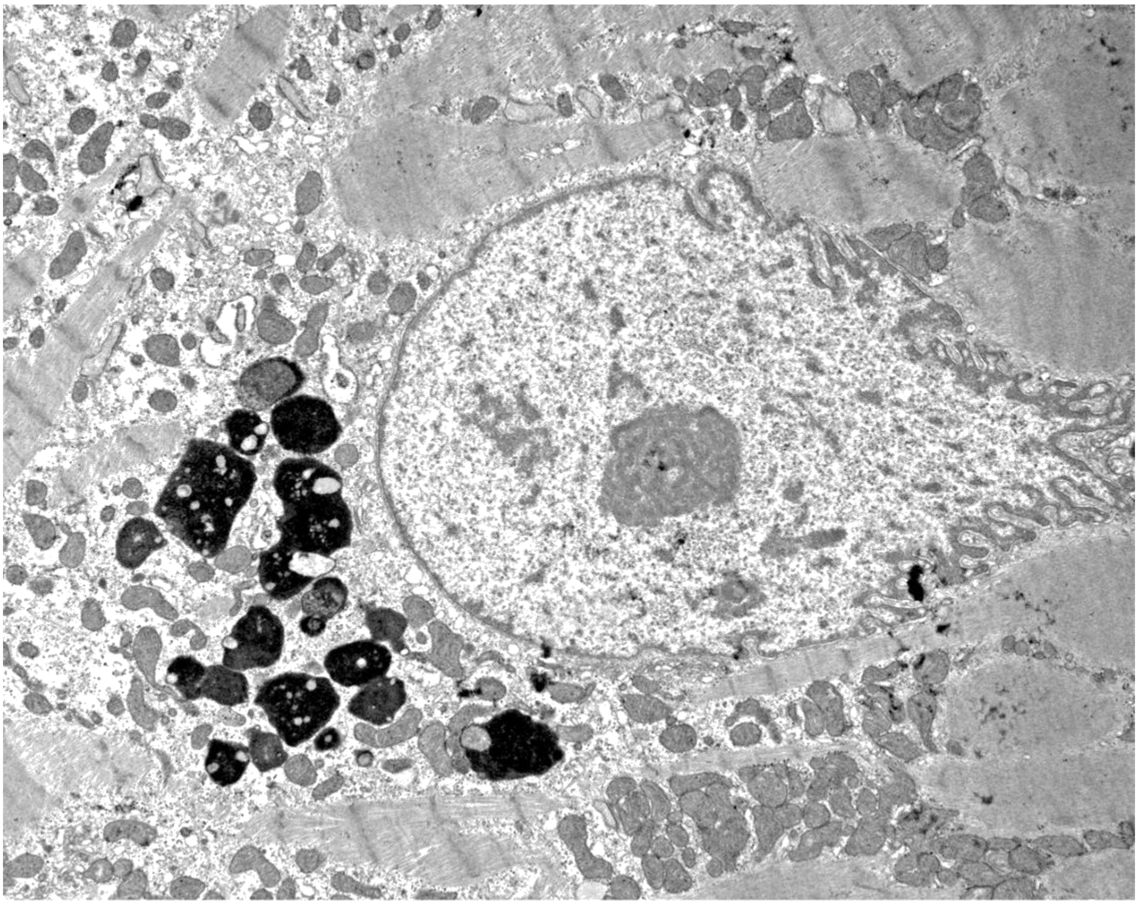

Supplemental Figure S11. Baseline transmission electron microscopy showing nuclear changes (chromatin fragmentation, abnormal nucleolonema) and cytoplasm changes (areas of myofibrillolysis, abnormal sarcomeres and accumulation of lipofuscins).

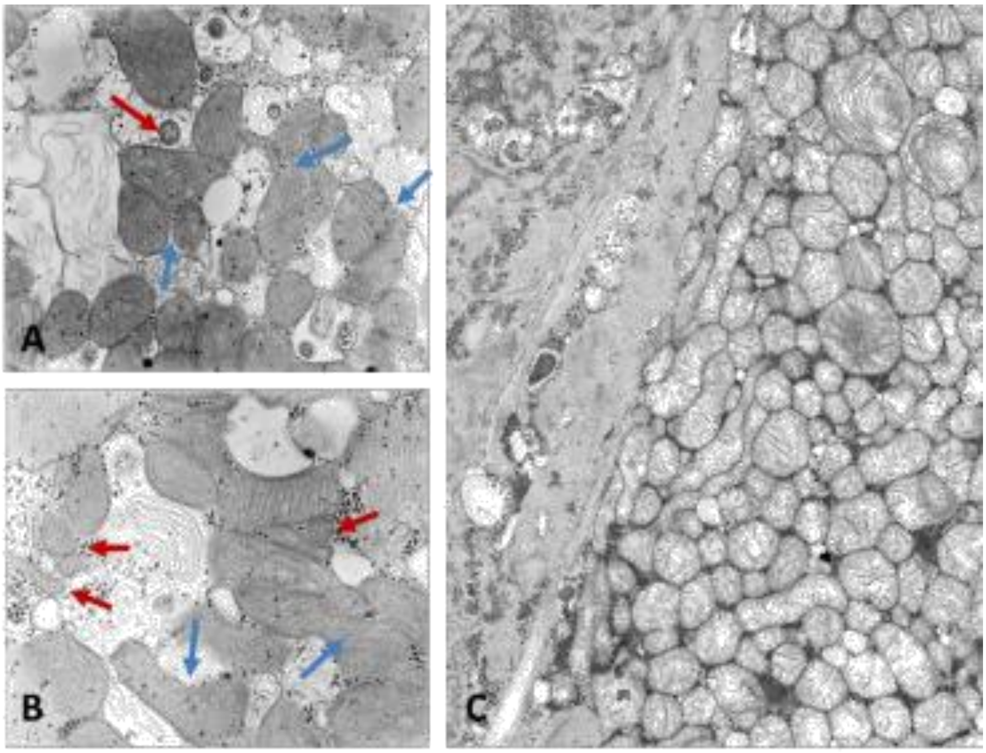

Supplemental Figure S12. Baseline transmission electron microscopy showing alterations of mitochondrial dynamics/conformation and small vesicles dilatation (sarcoplasmic reticulum, Golgi vesicles, lysosomes, mitochondria).

A-B. Evidences of altered fission/fusion dynamics, as shown by micro-mitochondria (red arrows) and fusing polymorphic/bizarre mitochondria (blu arrows).

C. A group of densely packed swollen mitochondria. The matrix is electron clear, while cristae are plate-like conformation, although sometimes fragmented.

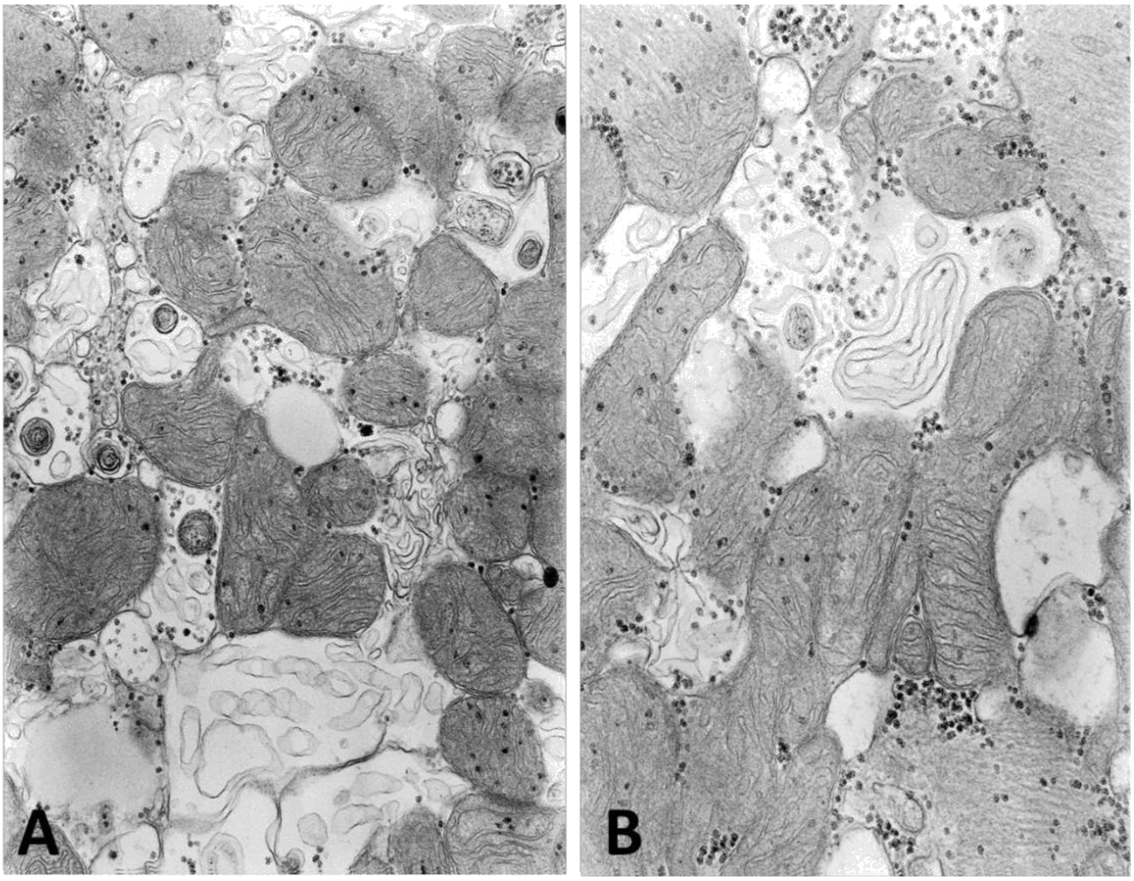

Supplemental Figure S13. Higher magnification of A and B electron panels of the figure 12.

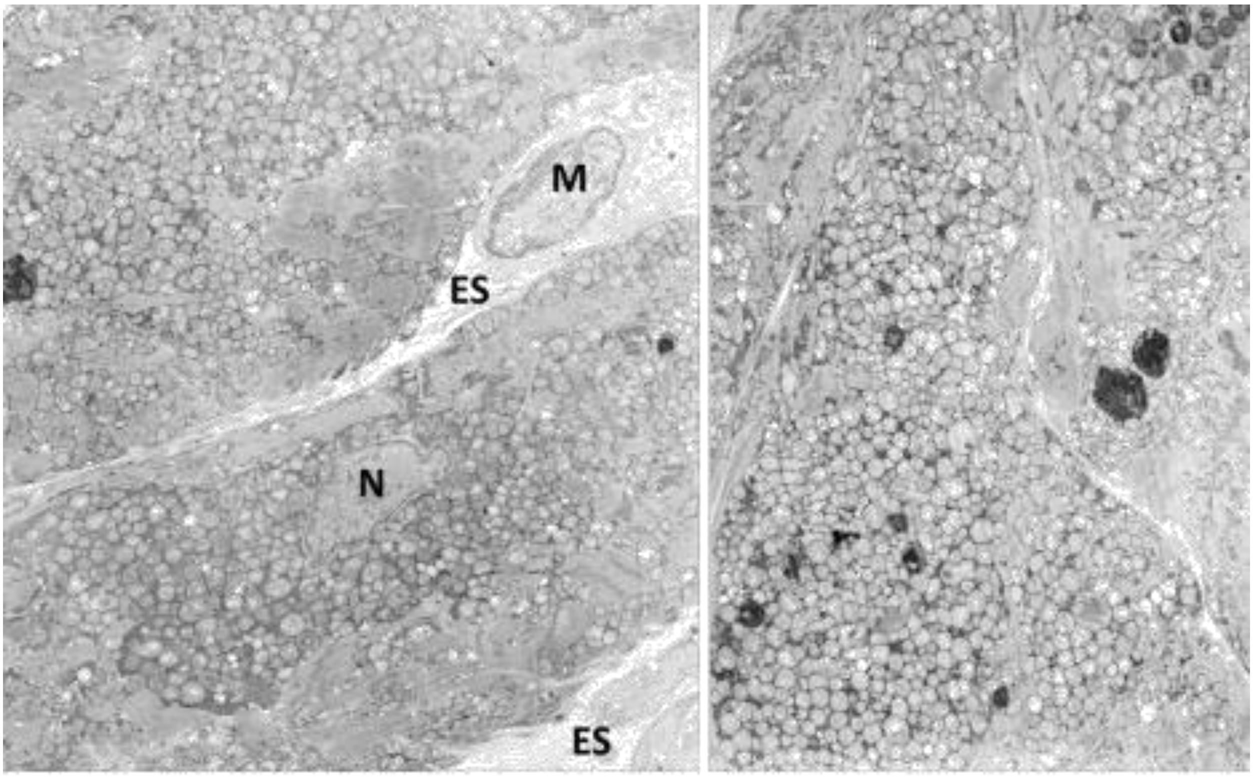

Supplemental Figure S14. Baseline transmission electron microscopy showing alterations of mitochondria.

Large groups of swollen mitochondria in a cytoplasm with reduced myofibrillar content and disorganized sarcomeres. Grouping of mitochondria may be responsible for an inefficient supply of high-energy phosphate molecules to the ATP-dependent functions, such as contraction. N = nucleus; ES = extracellular space; M = monocyte or large lymphocyte.

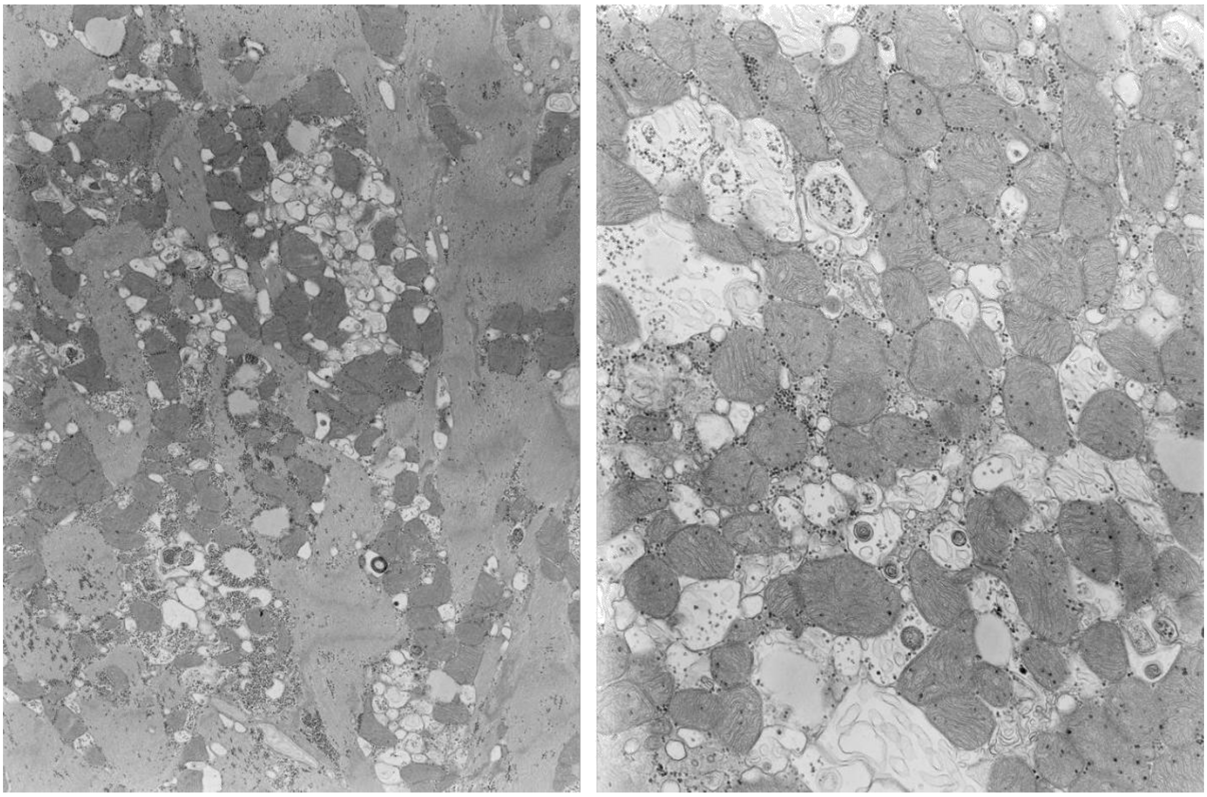

Supplemental Figure S15. Baseline transmission electron microscopy showing further details of dilated vesicles likely derived from sarcoplasmic reticulum, Golgi apparatus, lysosomes and other organelles.

The electron clear areas containing glicogen particles are either swollen cytosol or the lumen of a autophagocytic vacuole. Mitochondria are in orthodox/slightly swolle conformation.

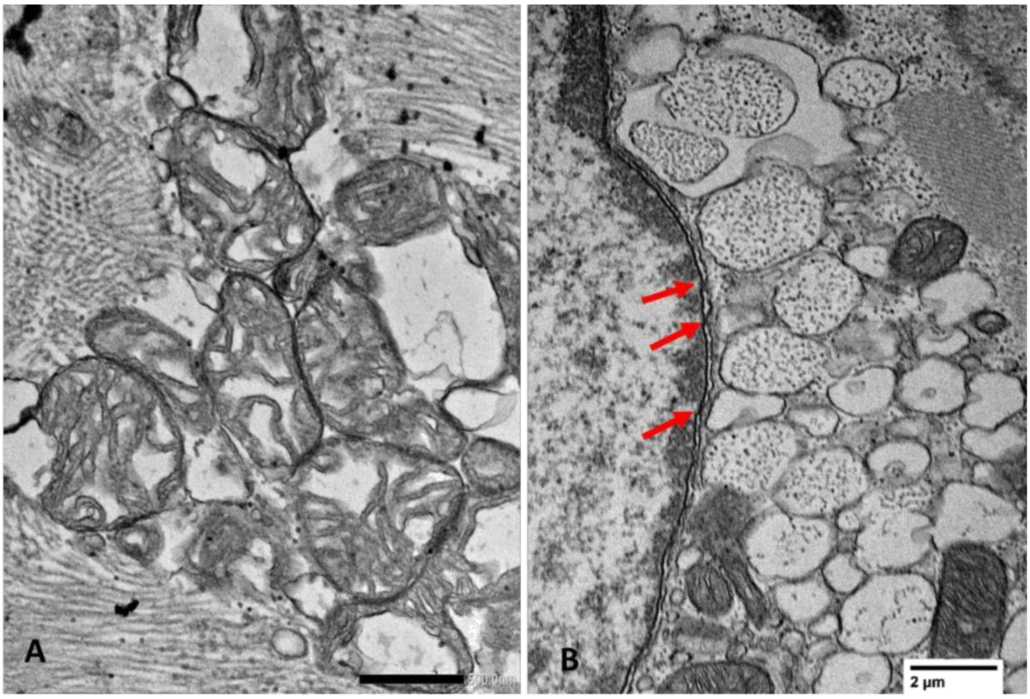

Supplemental Figure S16. Baseline transmission electron microscopy showing detail of highly swollen mitochondria and other organelles.

A. Detail of mitochondria: diluted and vesiculated matrix with irregular cristrae and damaged outer membrane

B. Detail small dilated vesicles of heterogeneous origin: phagosomes, autophagosomes, cyternae of sarcoplasmic reticulum (strictly interacting wit outer mitochondrial membrane) and, possibily, Golgi apparatus (close to nuclear membrane).

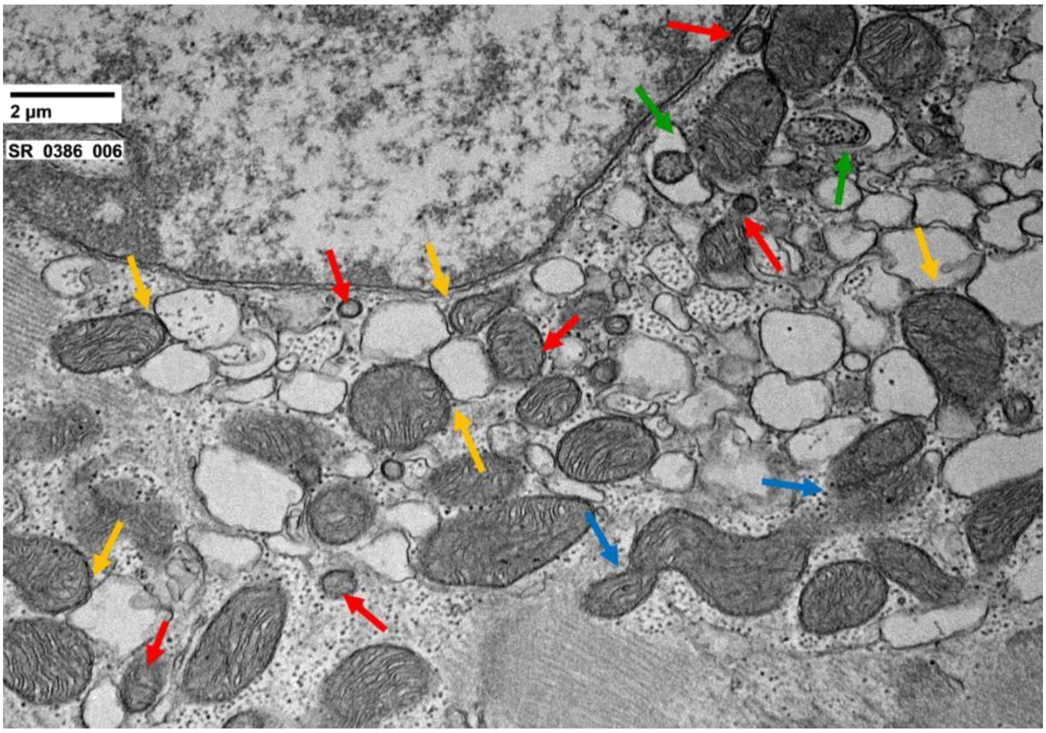

Supplemental Figure S17. Perinuclear region with small vesicles to that observed in the figure S16.

Electron microscopy showing a number of details of abnormal dynamics of mitochondria: micro-mitochondria (red arrows), bizarre fusion (blu arrows) close interaction with sarcoplasmic reticulum cysternae (orange arrows) and mitophagy (green arrows).

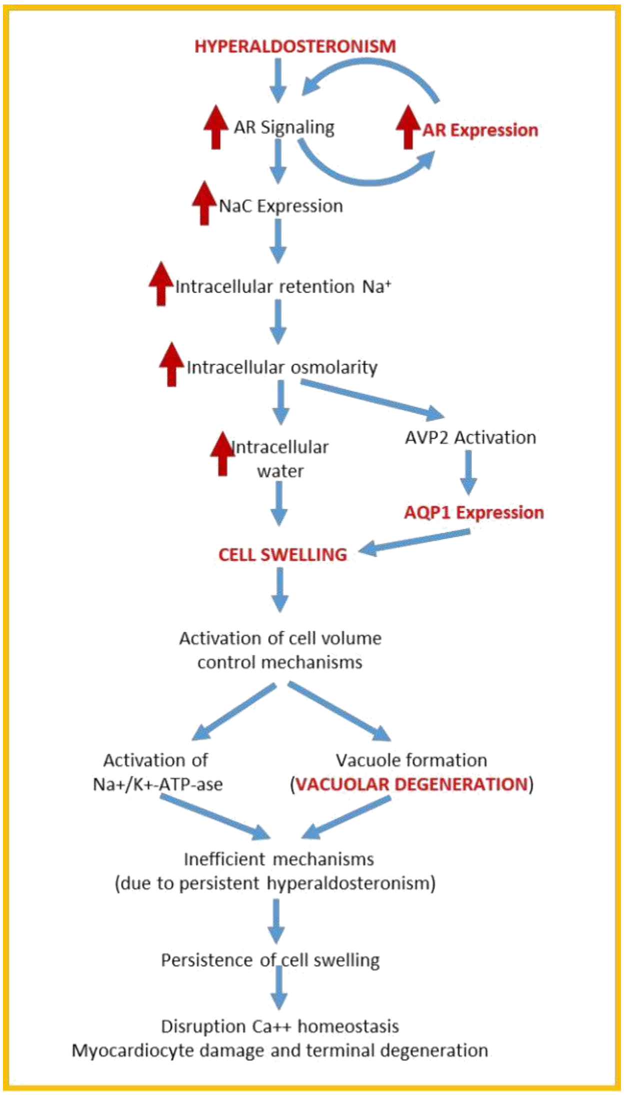

Supplemental Figure S18. An hypothetical description of the sequence triggered by aldosterone signaling leading to ion/water homeostasis disruption and to the myocardiocyte damage.

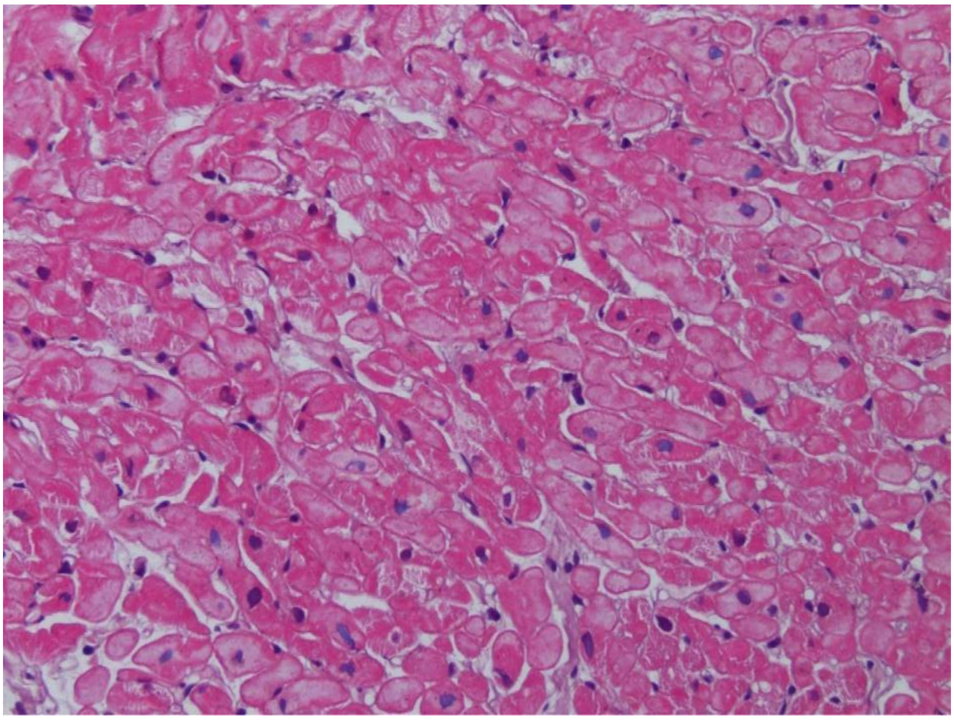

Supplemental Figure S19. Normal myocardium from EMB sample at histology.

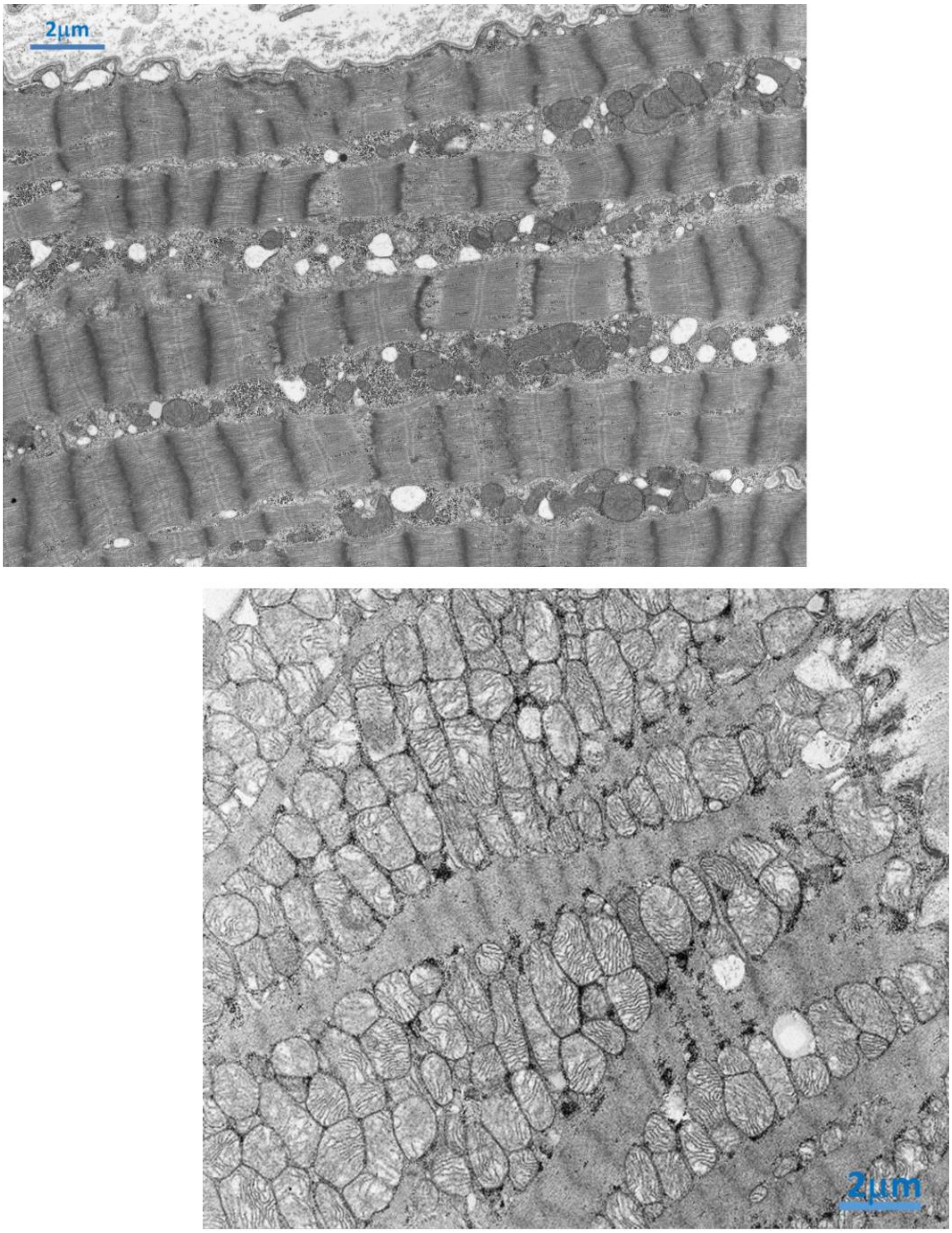

Supplemental Figure S20. Normal myocardium from a EMB sample at transmission electron microscopy
